# Relationship between fatty acid intake and aging: a Mendelian randomization study

**DOI:** 10.18632/aging.205674

**Published:** 2024-03-26

**Authors:** Yuhua Chen, Lian Yang, Kui Wang, Yu An, Yuping Wang, Ya Zheng, Yongning Zhou

**Affiliations:** 1 The First Clinical Medical College, Lanzhou University, Lanzhou 730000, China; 2 Department of Gastroenterology, The First Hospital of Lanzhou University, Lanzhou 730000, China; 3 Gansu Province Clinical Research Center for Digestive Diseases, The First Hospital of Lanzhou University, Lanzhou 730000, China; 4 Department of Radiology, Union Hospital, Tongji Medical College, Huazhong University of Science and Technology, Wuhan 430022, China; 5 Hubei Key Laboratory of Molecular Imaging, Wuhan 430022, China; 6 Department of Gastroenterology, Ruijin Hospital, School of Medicine, Shanghai Jiao Tong University Shanghai, Shanghai, China

**Keywords:** Mendelian randomization, aging, fatty acid, monounsaturated fatty acid, telomere length

## Abstract

Background: Observational studies have previously shown a possible link between fatty acids and aging-related diseases, raising questions about its health implications. However, the causal relationship between the two remains uncertain.

Methods: Univariable and multivariable Mendelian randomization (MR) was used to analyze the relationship between five types of fatty acids—polyunsaturated fatty acid (PUFA), monounsaturated fatty acid (MUFA), saturated fatty acid (SFA), Omega-6 fatty acid (Omega-6 FA), and Omega-3 fatty acid (Omega-3 FA) and three markers of aging: telomere length (TL), frailty index (FI), and facial aging (FclAg). The primary approach for Mendelian randomization (MR) analysis involved utilizing the inverse variance weighted (IVW) method, with additional supplementary methods employed.

Results: Univariate MR analysis revealed that MUFA, PUFA, SFA, and Omega-6 fatty acids were positively associated with TL (MUFA OR: 1.019, 95% CI: 1.006-1.033; PUFA OR: 1.014, 95% CI: 1.002-1.026; SFA OR: 1.016, 95% CI: 1.002-1.031; Omega-6 FAs OR=1.031, 95% CI: 1.006-1.058). PUFA was also associated with a higher FI (OR: 1.033, 95% CI: 1.009-1.057). In multivariate MR analysis, after adjusting for mutual influences among the five fatty acids, MUFA and PUFA were positively independently associated with TL (MUFA OR: 1.1508, 95% CI = 1.0724-1.2350; PUFA OR: 1.1670, 95% CI = 1.0497-1.2973, while SFA was negatively correlated (OR: 0.8005, 95% CI: 0.7045-0.9096).

Conclusions: Our research presents compelling evidence of a causal association between certain fatty acids and indicators of the aging process. In particular, MUFA and PUFA may play a role in slowing down the aging process, while SFAs may contribute to accelerated aging. These findings could have significant implications for dietary recommendations aimed at promoting healthy aging.

## INTRODUCTION

Aging in organisms is marked by declining biological functions and notable genetic and epigenetic changes [1]. Chronic diseases, frailty, and cognitive decline [2] constitute notable aging traits [3]. Accelerated aging leads to heightened disease and mortality risks, alongside diminished life expectancy and quality of life [4]. To evaluate accelerated aging, proxy indicators such as telomere length (TL), facial aging (FclAg), and frailty index (FI) play a pivotal role in evaluating biological age [5]. Efficiently identifying and managing influencing factors will contribute to averting premature mortality, prolonging healthy life expectancy, and enhancing overall quality of life.

Recent research has revealed that advancements in genetic engineering techniques have linked alterations in lipid metabolism with the process of aging and the development of age-related diseases [6]. Accumulation of lipids and compromised fatty acid utilization in organs are correlated with age-related pathophysiological traits. Variations in adipokine levels further contribute to the aging process by influencing systemic metabolism and inflammation [7]. There is evidence that dietary fatty acids may hasten aging [8, 9]. However, some studies have demonstrated that some fatty acids (e.g., Omega-3 [10, 11], non-long-chain saturated fatty acids [12, 13], and lower n-6:n-3 PUFA ratios [14], etc.) may mitigate aging. A definitive connection between fatty acids and the aging process has yet to be established [12].

**Figure 1:**
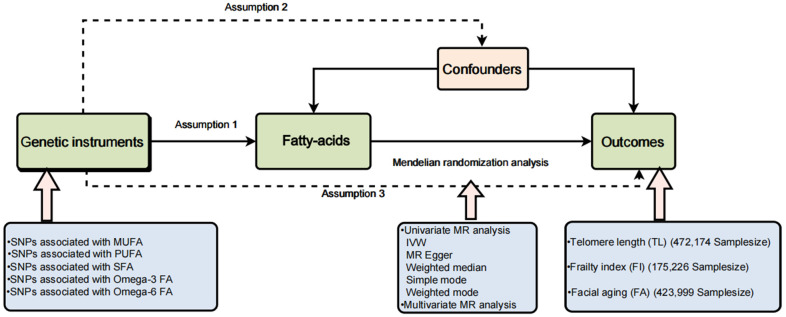
A brief flow chart of our Mendelian randomization study.

Due to evidential constraints, the possibility of reverse causality, and lingering confounding factors, observational studies have struggled to establish a causal link between fatty acids and the aging process [15]. Randomized controlled trials (RCTs) serve as a valuable tool for establishing causality in this context. However, it’s essential to note that RCTs come with substantial financial, temporal, and human resource costs, and some interventions may not be eligible or suitable for evaluation through this method [16]. In recent years, Mendelian randomization (MR) has gained prominence as a widely used and effective approach for causal inference. It leverages genetic variation, often in the form of single nucleotide polymorphisms (SNPs), as instrumental variables (IVs) to ascertain causal connections between exposure and outcome. This method effectively mitigates the confounding bias frequently encountered in traditional epidemiological studies [17]. There are three main hypotheses in MR. First hypothesis: There should be a significant correlation between lipid-related traits and genetic variants used as IVs. The second hypothesis states that no potential confounding variables should be connected to the IVs being used. The third hypothesis states that exposed genetic variants should only influence risk factors and no other potential pathways when determining the likelihood of an outcome. Figure 1 depicts the experiment’s study design.

In this Mendelian randomization (MR) investigation, we aimed to evaluate the causal relationship between fatty acids and the aging process. To achieve this, we analyzed summary-level data from genome-wide association studies (GWAS) encompassing various fatty acids, including saturated, monounsaturated, polyunsaturated, Omega-6, and Omega-3 fatty acids, as well as age proxy indicators such as telomere length (TL), frailty index (FI), and facial aging (FclAg), along with other related traits. Univariate MR analyses were designed to explore the relationship between individual fatty acids of each type and each indicator of aging, and multivariate MR analyses were employed to differentiate and compare the distinct effects of each fatty acid type on various aging indicators. To rule out the possibility of reverse causality, we performed reverse MR analyses to examine the effects of aging on five fatty acids.

## MATERIALS AND METHODS

### Data source

Genetic variants that exhibited significant associations with various fatty acid types (Saturated fatty acid, Monounsaturated fatty acid, Polyunsaturated fatty acid, Omega-3 fatty acid, and Omega-6 fatty acid) were extracted from a substantial Genetic Investigation of UK Biobank GWAS dataset, comprising a sample size of 114,999 individuals. Genetic variants linked to telomere length (TL) and facial aging (FclAg) were identified in two separate samples. The TL-associated sample comprised 472,174 individuals (216,187 males and 255,987 females) with an average age of 56.1±7.9 years. The FclAg-related sample included 423,999 individuals (194,391 males and 229,601 females) aged between 40 and 69 years. Telomere length in the mixed leukocyte population was measured in the UK Biobank using the multiplex quantitative polymerase chain reaction (qPCR) technique [18]. Facial aging (FclAg) was assessed using a questionnaire-based non-subjective perceived age. Participants’ responses were coded as 1, 0, or 0.5 depending on whether they thought they appeared younger, older, or similar to their age. FclAg is a variable that has an ordered categorical structure. Then, using a Taylor expansion series, a log odd ratio (OR) was used to convert linear scale statistics. OR > 1 denotes a higher likelihood of looking young [19]. A meta-analysis of GWAS carried out by Atkins et al., involving 164,610 participants (comprising 79,791 males and 84,819 females) with an average age of 64.1±2.8 years, as well as 10,616 participants (consisting of 5,039 males and 5,577 females) with an average age of 58.3±7.9 years, identified genetic variants significantly associated with the frailty index (FI) [20]. All GWAS sources used in the text can be found in Supplementary Table 1. This study exclusively utilized publicly accessible summary-level statistics, obviating the need for ethical approval.

### IV selection criteria

From the GWAS datasets, SNPs were chosen as IVs if they were found to be significantly associated with either exposures or outcomes (p<5×10^-8^, respectively). When linkage disequilibrium was detected in the candidate IVs (r^2^> 0.001), the variants within 1000 kb of other IVs with a stronger association were discarded. Palindromic SNPs are characterized by alleles that match nucleotides that form complementary base pairs at the DNA molecule, while intermediate allele frequencies are those that fall between 0.01 and 0.30 [21]. The process of IV selection should exclude palindromic SNPs. To meet the first Mendelian assumption, we calculated the R^2^ and F-statistic [22]. R^2^ was used as a tool of genetics to explain a portion of the trait’s variance. The calculation for R^2^ utilized the formula: R^2^ = 2 × (1 - MAF) × MAF × β^2^/ (SE^2^ × N), where β represents the effect size, SE is the standard error, N denotes the sample size, and MAF signifies the minimum allele frequency for each SNP. The F-statistic is a common metric for assessing the strength of instrumental variables and can be computed using the following formula [23]: F = beta^2^/se^2^. We deem the genetic variation used as a weak instrumental variable when the F-statistic falls below 10, which could introduce a potential bias in the results. F > 10 was considered to be sufficient strength, whereas an F-statistic of 10 indicates “weak instruments”.

### Statistical analysis

In the context of univariate Mendelian randomization (MR) analyses, we employed the inverse variance weighted (multiplicative random effects) (IVW-MRE) method for conducting a two-sample MR analysis. This approach was utilized to evaluate the potential causal relationships between exposures, including SFA, MUFA, PUFA, Omega-3 fatty acid, and Omega-6 fatty acid, and outcomes such as TL, FclAg, and FI. Additionally, for further analysis, we employed the weighted median, weighted mode, simple mode and MR Egger methods [24]. We also conducted multivariate inverse variance weighted (IVW) analysis to assess the independent causal effects of the relevant fatty acid traits on aging proxy indicators. The results of the Mendelian randomization (MR) analysis have been presented as odds ratio (OR) with 95% confidence intervals (CIs). We used MR Egger method and MR Pleiotropy RESidual Sum and Outlier (MR-PRESSO) tests to assess the potential multiplicity of effects, with a p-value greater than 0.05 indicating no evidence of the multiplicity of effects. In addition, we performed sensitivity analyses to validate and enhance the reliability and stability of the results. These analyses encompassed: 1. Heterogeneity Test: This included Cochrane’s Q test, MR-Egger test, and the Inverse Variance Weighted test to assess heterogeneity among the studies. 2. Pleiotropy Test: We conducted tests such as the MR Egger intercept test and MR-PRESSO global test to assess and account for pleiotropy, which is the phenomenon where a single genetic variant affects multiple traits. 3. Leave-One-Out Test: We also carried out a leave-one-out test, systematically excluding one study at a time, to evaluate the impact of individual studies on the overall results. These sensitivity analyses were implemented to ensure the robustness of our findings [24–27].

We employ the Bonferroni correction for multiple comparisons in our univariate MR analyses, in our analysis, p-values falling within the range of 0.05 to 0.0033 were considered as suggestive evidence of potential causality, whereas p-values less than 0.0033 (calculated as 0.05 divided by the total number of tests, which is 5 times 3) were viewed as statistically significant evidence of causality. Additionally, p-values less than 0.05 were considered statistically significant proof of causality. P < 0.05 was taken into account statistically significant proof of causation because the Bonferroni method was not applicable to multivariate MR analyses. The current study was carried out using the R software (version 4.2.1).

### Data availability statement

The datasets of GWASes are available from the website: https://gwas.mrcieu.ac.uk/.

## RESULTS

### Univariate MR

#### Fatty acid and TL

**Figure 2:**
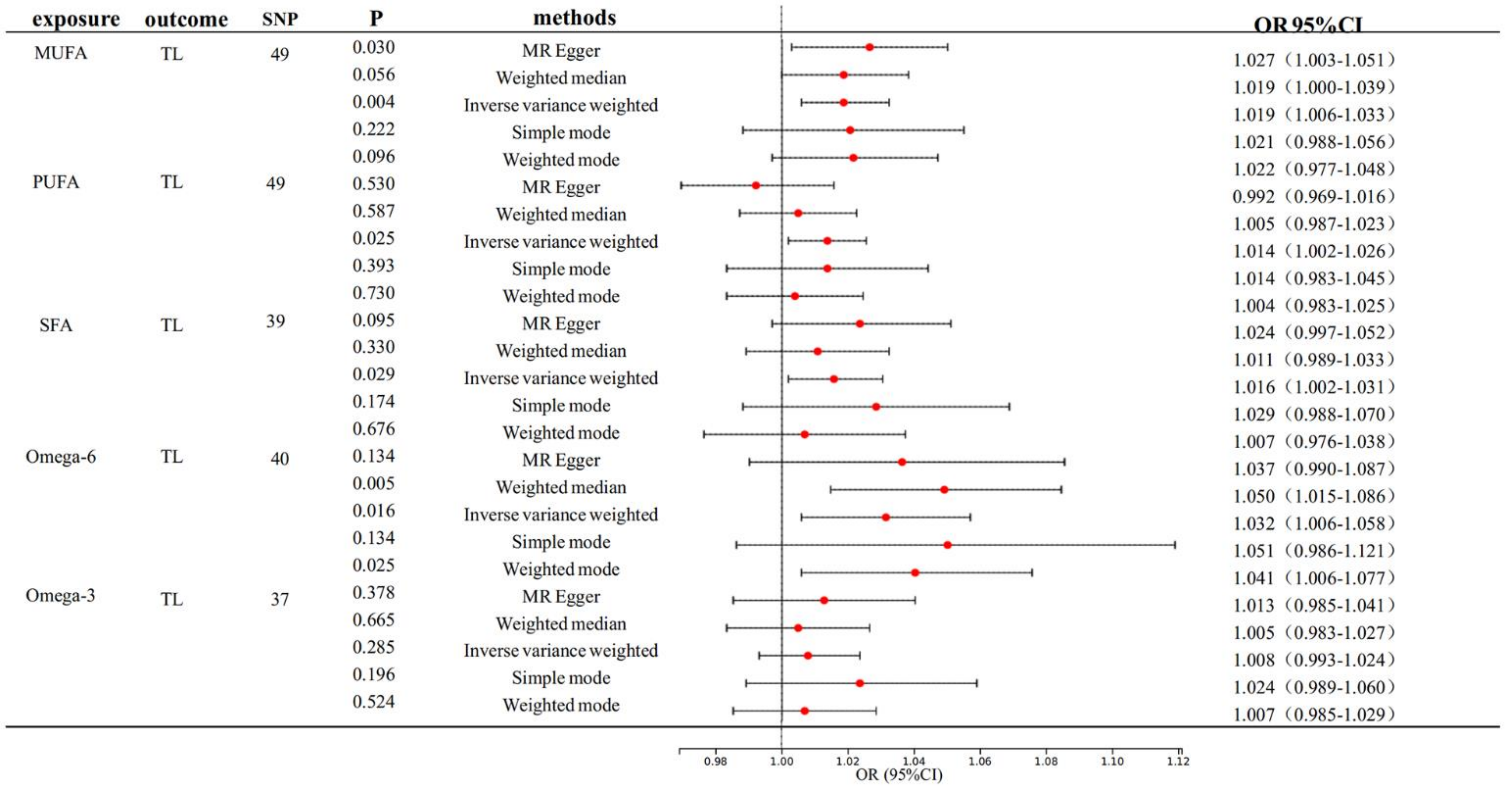
**Mendelian randomization analysis of the effect of FA on TL.** FA, fatty acid, TL, telomere length.

Figure 2 depicts the primary findings of the MR analysis. The IVW-MRE method revealed significant causal relationships between genetically predictive MUFA on TL. (OR: 1.019, 95% CI= 1.006-1.032, p<0.0033). In addition, we also found a suggestive causal relationship between PUFA (OR: 1.014, 95% CI= 1.002-1.026, p<0.05), SFA (OR: 1.016, 95% CI= 1.002 -1.031, p<0.05) and Omega-6 fatty acid (OR: 1.032, 95% CI= 1.006-1.058, p<0.05) respectively on TL. We can see that their F-statistics are all greater than 10, indicating that the instrumental variables are appropriately selected and are strongly correlated variables.

We also performed TL reverse association analyses on MUFA, PUFA, and SFA, which did not reveal any reverse causality (Supplementary Table 2). Our study incorporated sensitivity analysis, wherein leave-one-out tests underscored the efficacy of the associations between fatty acids and telomere length (TL). Nevertheless, heterogeneity was observed in the analysis of TL with MUFA, PUFA, SFA, and Omega-6 fatty acid (P < 0.01). Notably, the MR-Egger’s test revealed a p-value of 0.045, which is less than the significance threshold of 0.05, indicating horizontal multiplicity of PUFA for TL (Supplementary Table 3 and Supplementary Figure 1).

#### Fatty acid and FI

**Figure 3:**
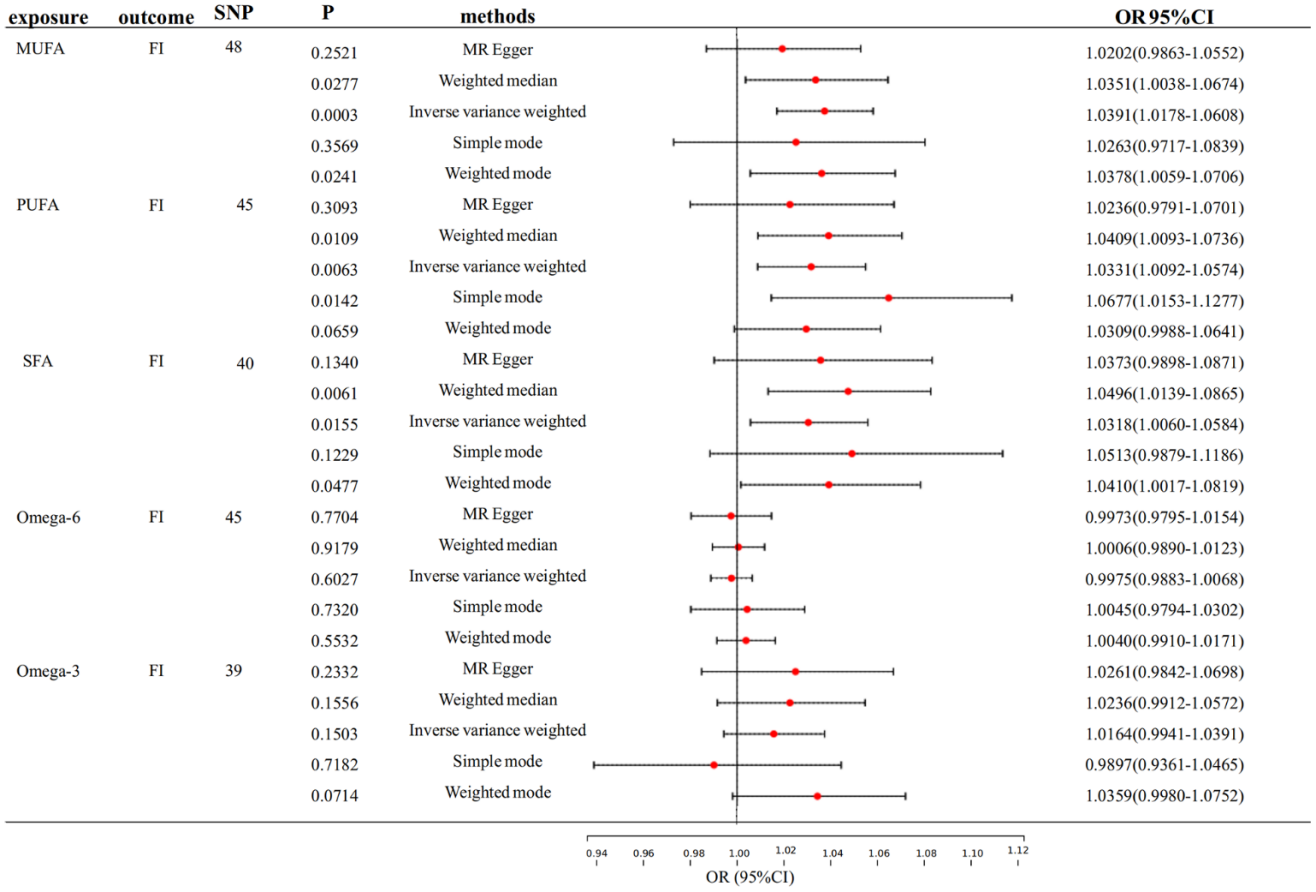
**Mendelian randomization analysis of the effect of FA on FI.** FA, fatty acid, FI, frailty index.

Figure 3 shows the primary findings of the MR analysis. The IVW-MRE method revealed significant causal relationships between genetically predictive MUFA and PUFA on FI. (OR: 1.039, 95% CI= 1.018-1.061, p <0.0033; OR: 1.033, 95% CI= 1.009-1.057, p <0.0033). Also, we found a suggestive causal relationship between SFA and FI (OR: 1.032, 95% CI= 1.006-1.058, p<0.05). Besides, its F-statistic is also greater than 10. Similarly, we found no pleiotropy or heterogeneity, nor any anomalous outliers in MUFA, PUFA and SFA on FI (Supplementary Table 3 and Supplementary Figure 1). While heterogeneity and pleiotropy were identified in the MR analyses of Omega-6 and FI, the results of the sensitivity analyses were non-significant due to the absence of a discernible causal link between the two.

We also performed FI reverse association analyses on MUFA, PUFA, and SFA, However, we found a reverse causality association of FI on MUFA, and FI on SFA with a robust result (Supplementary Table 2). Thus, there is bi-directional causality in the relationship of FI with MUFA and SFA, which is dropped due to the instability of the results.

#### Fatty acid and FclAg

The results proved that there was no causal association between any kind of aging proxy indicators and FclAg. Heterogeneity and pleiotropy were observed between PUFA and FA, as well as between Omega-6 fatty acid and FclAg. However, given the lack of significance in the primary outcome using the Inverse Variance Weighting (IVW) method, we can dismiss this finding. Furthermore, leave-one-out tests revealed that the relationships were robust, as indicated in Supplementary Table 3 and Supplementary Figure 1.

### Multivariate analysis

**Figure 4:**
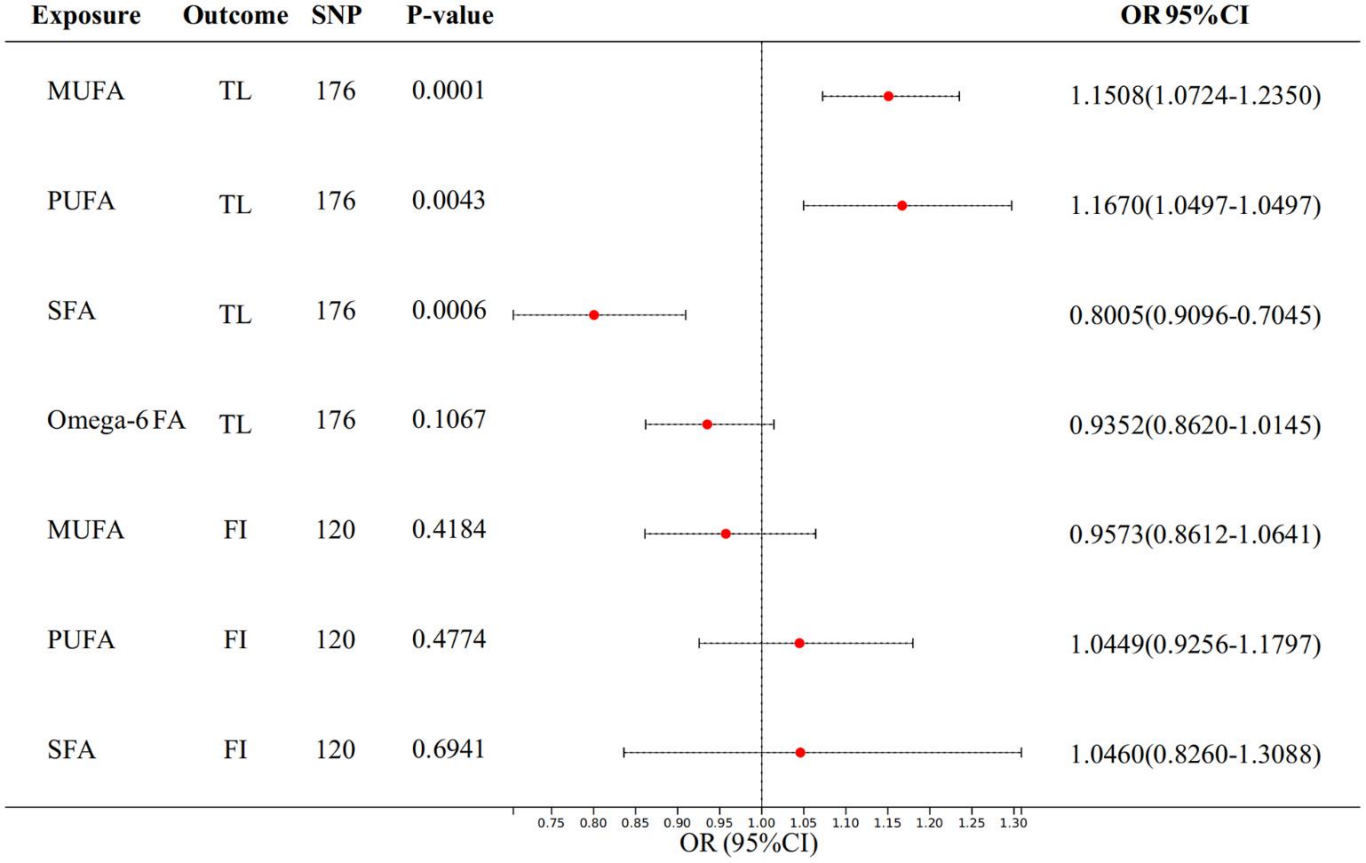
The result of multivariate analysis of FA on telomere length and frailty index.

We used multivariate MR analyses to further assess the relationships between fatty acids (FA) and proxy indicators of aging. We analyzed the univariate results in the previous section again by adjusting the variables with positive results for each other. For TL, after adjusting for MUFA, PUFA, SFA, and Omega-6 fatty acid at once, the causal association of MUFA (p = 0.0001) PUFA (p = 0.0043) and SFA (p = 0.0006) remained significant (Figure 4). And we can see that MUFA (OR: 1.1508, 95% CI = 1.0724-1.2350) and PUFA (OR: 1.1670, 95% CI = 1.0497-1.2973) are positively correlated with TL, while SFA is negatively correlated with TL (OR: 0.8005, 95% CI = 0.7045-0.9096). The significance is more significant than when it is univariate.

However, causal relationships between MUFA, PUFA, SFA, and FI all become uncorrelated after adjusting for each other.

## DISCUSSION

In this study, we investigated how five types of fatty acids affect aging. Our main findings suggest that MUFA and PUFA have a positive effect on TL, a marker related to aging. In contrast, SFA appears to shorten TL. These relationships remained even after adjustments were made for other variables. Thus, MUFA and PUFA help alleviate aging (TL increased). In addition, SFA accelerates aging (TL decreased).

It is well known that telomeres are strongly associated with aging characteristics [28]. A commonly used clinical tool for frailty, which reflects increased vulnerability to adverse health outcomes in individuals of the same actual age, is the frailty index, which is also a marker of aging [29]. Additionally, skin aging can be a visible sign of aging [30]. Evaluating accelerated aging through the measurement of changes in biological age proxy indicators like TL, FI, and FclAg is of paramount importance. This approach aids in identifying contributing factors to accelerated aging and devising interventions to combat aging-related diseases, including but not limited to cardiovascular disease, cancer, arthritis, type 2 diabetes, and Alzheimer’s disease [7].

Additionally, due to their potential anti-inflammatory and antioxidant effects, fatty acid consumption is associated with many diseases [31–33]. It has been demonstrated that the pathophysiological phenotypes of aging are associated with lipid buildup and impaired fatty acid utilization in organs. The aging process is accelerated by changes in adipokine levels, which control changes in systemic metabolism and inflammation [34, 35]. However, it is challenging to pinpoint and affect the genes that cause accelerated aging. A technique for determining causality based on genetic variation that can be used to investigate accelerated aging is MR analysis. In this study, we collected extensive genome-wide association study (GWAS) data on telomere length (TL), frailty index (FI), and fatty acids (FA) from the UK Biobank. Subsequently, through MR analysis, we discovered that MUFA and PUFA were linked to an increase in TL after adjusting for MUFA, PUFA, FA, and Omega-6 fatty acid, while SFA was associated with decreased TL. Because a decrease in TL, an increase in FI, and an increase in FclAg may be indicators of accelerated aging, the study’s findings suggest that supplementation with MUFA and PUFA may be causally related to aging alleviation, whereas supplementation with SFA will accelerate aging. Overall, MUFA and PUFA appear to slow down aging, whereas SFA appears to increase the risk of premature aging. This implies that the structure of fatty acid intake is crucial for slowing down the effects of aging. Additionally, this encourages us to eat more foods containing unsaturated fatty acids and less saturated fatty acid foods, thereby easing the progression of aging and further reducing the incidence of aging-related diseases.

This study’s key point is to investigate the causal impact of fatty acids on the aging process. The accurate detection and monitoring of dietary fatty acid (FA) intake values can be challenging, which represents a limitation in traditional epidemiological studies. However, MR analyses offer a strength in this regard, as they can provide valuable insights into the causal relationships involving fatty acids without relying on self-reported intake values. In conventional epidemiological studies, the relationship between fatty acids (FA) and aging can be heavily influenced by diet and other lifestyle habits, and the effect of diet and other habits on fatty acids levels is difficult to determine. Our study, on the other hand, looked into the risk of aging variability in fatty acids levels determined by genetic variation. By introducing instrumental variables, MR analyses were designed to avoid traditional confounders and to monitor their interference through sensitivity analyses. Thus, it is possible to balance the effects of diet and other habits without affecting the outcomes.

Fatty acids, a significant part of the Western diet, and their effects on aging have been thoroughly researched. It is recognized that the Mediterranean diet is correlated with improved cognitive function, enhanced brain health, and a diminished risk of attention deficit disorder [36]. These favorable effects are believed to stem, at least partially, from the elevated levels of Omega-3 fatty acids, present in the Mediterranean diet [37]. According to studies, MUFA may lower the risk of dementia [38, 39]. In a sample of 1,223 individuals, they found unsaturated fatty acids (MUFA, PUFA) may attenuate the risk associated with mild cognitive impairment (MCI), The OR (95% CI) for total PUFA was 0.44 (0.29-0.66; p for trend = 0.0004), for Omega-6 fatty acids was 0.44 (0.30-0.67; p for trend = 0.0004), for Omega-3 fatty acids was 0.62 (0.42-0.91; p for trend = 0.012), and for (MUFA+PUFA) was 0.56 (0.38-0.83; p for trend = 0.01) [40]. Significantly, the sustained supplementation of DHA over 24 months demonstrated enhanced cognitive function in patients with MCI, accompanied by alterations in blood Aβ-related biomarkers. These modifications encompassed reduced levels of Aβ-42 and APP mRNA expression, alongside increased expression of Beclin-1, LC3-II, and LC3-II mRNA [41]. Long-chain SFA, MUFA, and PUFA did not significantly correlate with aging in a study of the relationship between total dietary food fat and type and peripheral leukocyte telomere length was measured in a cohort of 4,029 healthy postmenopausal women who participated in the Women’s Health Initiative study, but short-to-medium-chain saturated fatty acids (SMSFAs; aliphatic tails of 12 carbons) did. In addition, there are many studies demonstrating that telomere length increases as the n-6:n-3 ratio decreases [8, 10, 14]. Supplementation of dietary Omega-3 fatty acid may also be beneficial in reducing aging [42–44]. Furthermore, a recent MR study, which investigated the association between different fatty acids types and FI, found that, while there wasn’t a strong correlation observed with MUFAs or PUFAs, plasma stearic acid levels, which is one of the saturated fatty acids, showed a statistically significant association with a higher FI (β = 0.178; 95% CI = 0.050-0.307; p = 0.007) [45]. The results of our MR analysis are similar to the results of most existing high-quality studies that tend to suggest that unsaturated fatty acid-rich diets slow down aging and saturated fatty acid-rich diets and promote aging, and we genetically performed causal associations and multivariate analyses to ensure that the results are reliable and valid.

The aging process is accompanied by a persistent inflammatory state within the central nervous system. Significantly, astrocytes enriched with DHA exhibited diminished responsiveness to interleukin-1β (IL-1β). This reduction was mediated through the activation of inhibitory nuclear factor κB (NFκB) and activator protein 1 (AP-1) transcription factors. Furthermore, there were decreased levels of inducible nitric oxide synthase (iNOS) and cyclooxygenase-2 (COX-2), as well as a decline in the release of the pro-inflammatory cytokines tumor necrosis factor (TNF) and interleukin-6 (IL-6) [46]. Several PUFAs, such as arachidonic acid, play critical roles in cell signaling pathways, controlling a variety of bodily functions such as vasodilation, inflammation, and cell growth [47]. Other essential PUFAs are also involved in a wide range of cellular processes [39]. Indeed, Omega-3 fatty acids are recognized for their potent anti-inflammatory properties, achieved by facilitating the resolution phase of inflammation [48]. Besides, oxidative stress arises from an imbalance between free radicals and antioxidants in the body [49]. Free radicals, recognized as reactive oxygen species (ROS) or reactive nitrogen species (RNS), can instigate oxidative damage by disrupting cell membranes, DNA, and other vital cellular components [50]. Furthermore, a high-fat diet rich in DHA (45 kcal% fat, 1% DHA, W/W) prevented hippocampal insulin resistance induced by the high-fat diet in aged rats, leading to cognitive enhancement [51]. These beneficial effects can be attributed to the improvement of glucose homeostasis, alleviation of hippocampal neuroinflammation, and reduction of oxidative stress. Nuclear receptors (NRs) belong to the ligand-activated transcription factor superfamily, regulating the expression of numerous genes associated with various biological processes, energy regulation, and lipid metabolism in response to environmental and dietary cues [52]. Significantly, supplementation with DHA (403 mg/kg) and EPA (395 mg/kg) in rats has demonstrated the mitigation of age-related declines in RXRγ, calmodulin-dependent protein kinase II (CAMKII), protein kinase B (PKB/AKT), and recombinant extracellular signal-regulated kinase 1 (ERK1). This ultimately leads to enhanced spatial memory [53]. There are some recent examples of the life extension effects of lipid-related interventions [54]. Caenorhabditis elegans lifespan has increased as a result of several genetic modifications related to lipids [55]. It’s intriguing to highlight that Han et al. established a connection between chromatin remodeling and lipid metabolism during the aging process in Caenorhabditis elegans. Overexpression of dietary MUFAs or adipose-7 in the gut is sufficient to extend lifespan [56]. Chromatin modifiers called sirtuin histone deacetylases connect aging and metabolism. Sirtuins control vital metabolic processes, such as lipid metabolism and longevity, by deacetylating histone and non-histone proteins [57, 58]. To manage cognitive decline, GRADE (Grades of Recommendation Assessment Development and Evaluation) has advised a high intake of mono- or poly-unsaturated fatty acids in combination with a low intake of saturated fatty acids (1B) [59]. Among the possible mechanisms, DHA has been the most studied as a representative of the fatty acid-aging relationship, and the related possibilities it exposes deserve further exploration and validation. Overall, fatty acids affect the aging process through several complex cellular and molecular mechanisms, which are essential for gaining insight into the interrelationships between diet, metabolic health, and longevity. Future studies should further unravel the molecular mechanisms of different types of fatty acids and their long-term effects on overall health and longevity.

The following are the strengths of this MR study. The research design was grounded in three fundamental instrumental variable assumptions and adhered to the checklist for conducting MR investigations [60]. As a result, the study’s conclusions were reasonable and trustworthy. Second, because all of the data from the large-scale GWASs came from people with European ancestry, population stratification’s bias was avoided. Third, to evaluate the consistency of causal effects, five different MR analysis techniques were employed. We applied a multivariate adjustment methodology, demonstrating once again the robustness of our primary findings. Despite the large sample size, our study exhibits several limitations. We employed five Mendelian Randomization (MR) analysis methods in a comparative manner to ascertain the consistency and precision of each outcome’s directionality, but it’s important to note that we cannot entirely eliminate the possibility of residual bias, as this is a recognized limitation of Mendelian randomization (MR) studies. We must acknowledge the presence of pleiotropy and heterogeneity in certain outcomes obtained from this study. While the majority of these outcomes exhibit a negative trend, the significance of the discussion is somewhat limited. Despite the absence of outliers, the challenge in rectifying them may stem from the existence of unidentified confounding factors, a consequence of the current restricted understanding of both aging and fatty acids. Furthermore, MR studies frequently look at the long-term effects of risk factors on outcomes because it is difficult to determine the causal effects of different stages of disease development. The gender difference in fatty acid intake and accelerated aging was not possible in this study due to the absence of gender-stratified genome-wide association study (GWAS) data for fatty acids or aging at this time. Third, we must acknowledge that compared to RCT, MR analysis is less causality-suggestive, and it still needs to be complemented and supported by high-quality RCT evidence.

## CONCLUSIONS

Drawing from the results of the current study, MUFA and PUFA demonstrate potential in mitigating the aging process, as evidenced by an increase in telomere length (TL). Conversely, SFA intake appears to expedite aging, leading to a reduction in TL. Consequently, it is imperative to underscore the significance of augmenting unsaturated fatty acid consumption while reducing saturated fatty acids (SFA) intake as a preventative measure against accelerated aging. Interventions targeting lipid-related factors have the potential to effectively decelerate the aging trajectory and ameliorate age-associated ailments.

## Supplementary Material

Supplementary Figures

Supplementary Table 1

Supplementary Table 2

Supplementary Table 3
