# Supplementary material for: Relationship between fatty acid intake and aging: a Mendelian randomization study
Source: Aging (Albany NY). 2024 Mar 26;16(6):5711–39. doi: 10.18632/aging.205674 (PMC11006485; doi:10.18632/aging.205674)
Supplement: Supplementary Figures [file aging-16-205674-s001.pdf]

## SUPPLEMENTARY FIGURES

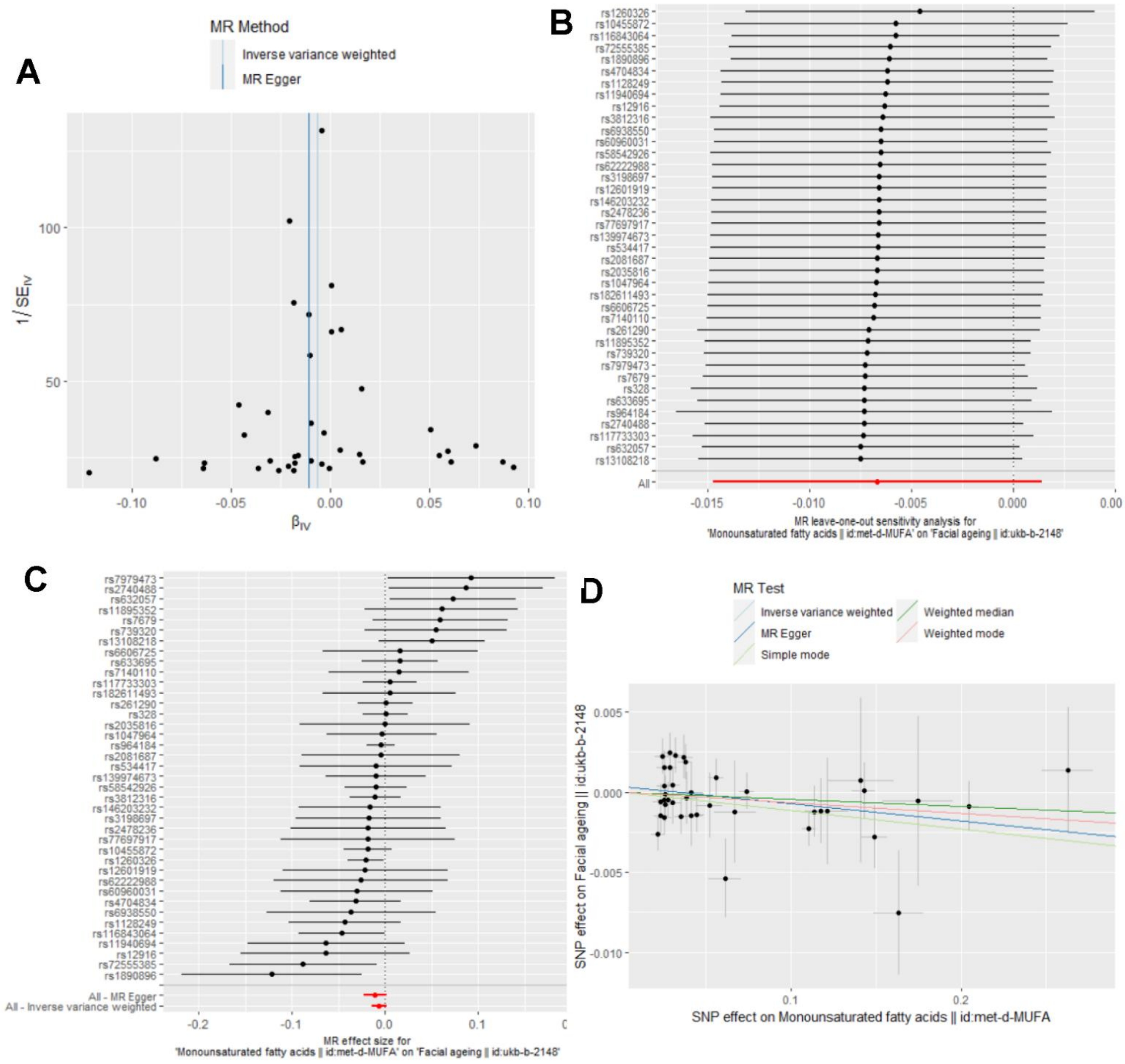


MUFA ON FA

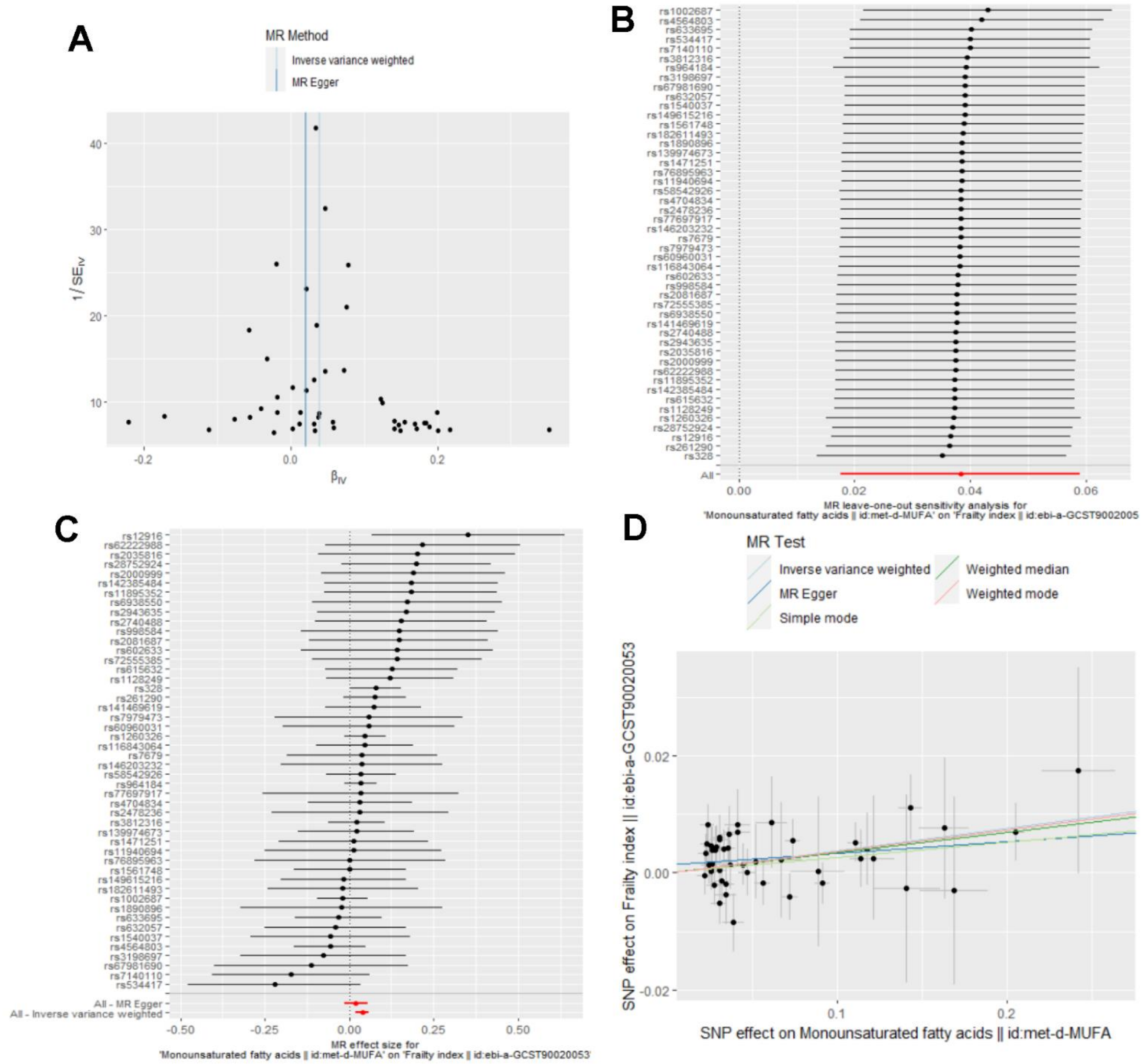
A
MR Method
Inverse variance weighted
MR Egger
1/SE_IV
β_IV
B
MR leave-one-out sensitivity analysis for
'Monounsaturated fatty acids || id:met-d-MUFA' on 'Frailty index || id:ebi-a-GCST9002005
C
All - MR Egger
All - Inverse variance weighted
MR effect size for
'Monounsaturated fatty acids || id:met-d-MUFA' on 'Frailty index || id:ebi-a-GCST90020053'
D
MR Test
Inverse variance weighted
MR Egger
Simple mode
Weighted median
Weighted mode
SNP effect on Frailty index || id:ebi-a-GCST90020053
SNP effect on Monounsaturated fatty acids || id:met-d-MUFA

MUFA ON FI

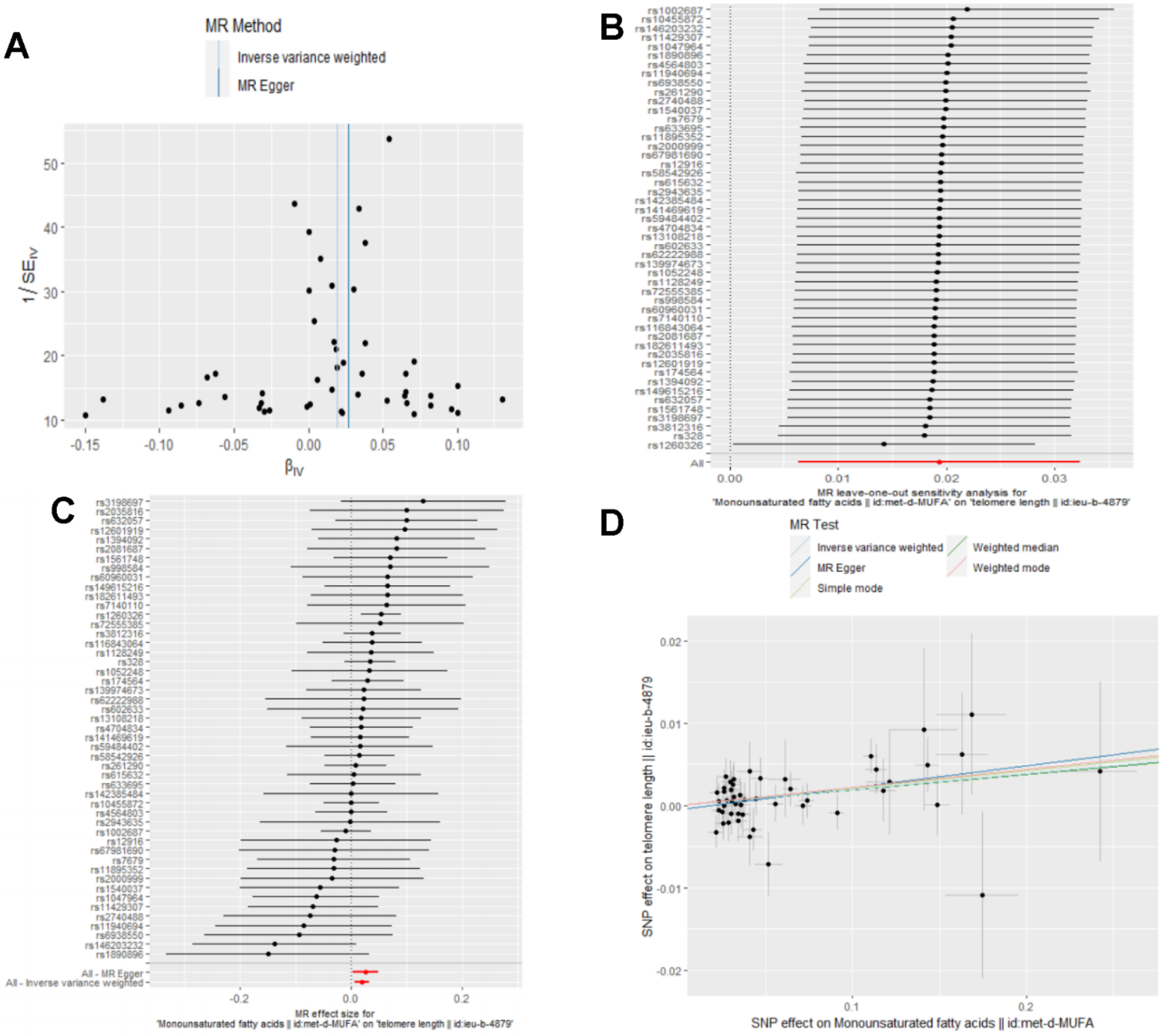
A
MR Method
Inverse variance weighted
MR Egger
1/SE_IV
β_IV
B
MR leave-one-out sensitivity analysis for
'Monounsaturated fatty acids || id:met-d-MUFA' on 'telomere length || id:ieu-b-4879'
C
All - MR Egger
All - Inverse variance weighted
MR effect size for
'Monounsaturated fatty acids || id:met-d-MUFA' on 'telomere length || id:ieu-b-4879'
D
MR Test
Inverse variance weighted
MR Egger
Simple mode
Weighted median
Weighted mode
SNP effect on telomere length || id:ieu-b-4879
SNP effect on Monounsaturated fatty acids || id:met-d-MUFA

MUFA ON TL

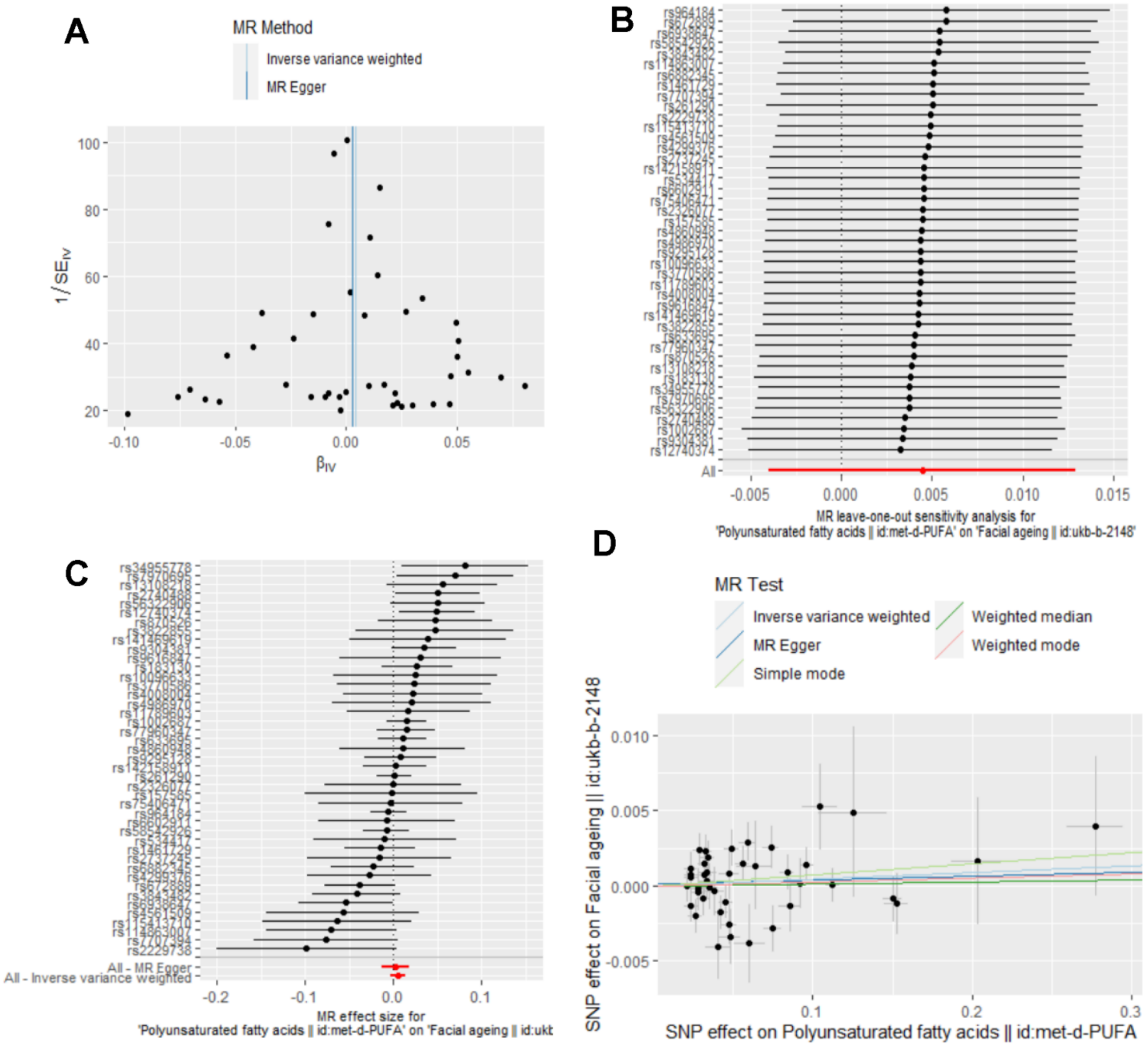
A
MR Method
Inverse variance weighted
MR Egger
1/SE_IV
β_IV
B
MR leave-one-out sensitivity analysis for
'Polyunsaturated fatty acids || id:met-d-PUFA' on 'Facial ageing || id:ukb-b-2148'
All
C
All - MR Egger
All - Inverse variance weighted
MR effect size for
'Polyunsaturated fatty acids || id:met-d-PUFA' on 'Facial ageing || id:ukb
D
MR Test
Inverse variance weighted
MR Egger
Simple mode
Weighted median
Weighted mode
SNP effect on Facial ageing || id:ukb-b-2148
SNP effect on Polyunsaturated fatty acids || id:met-d-PUFA

PUFA ON FA

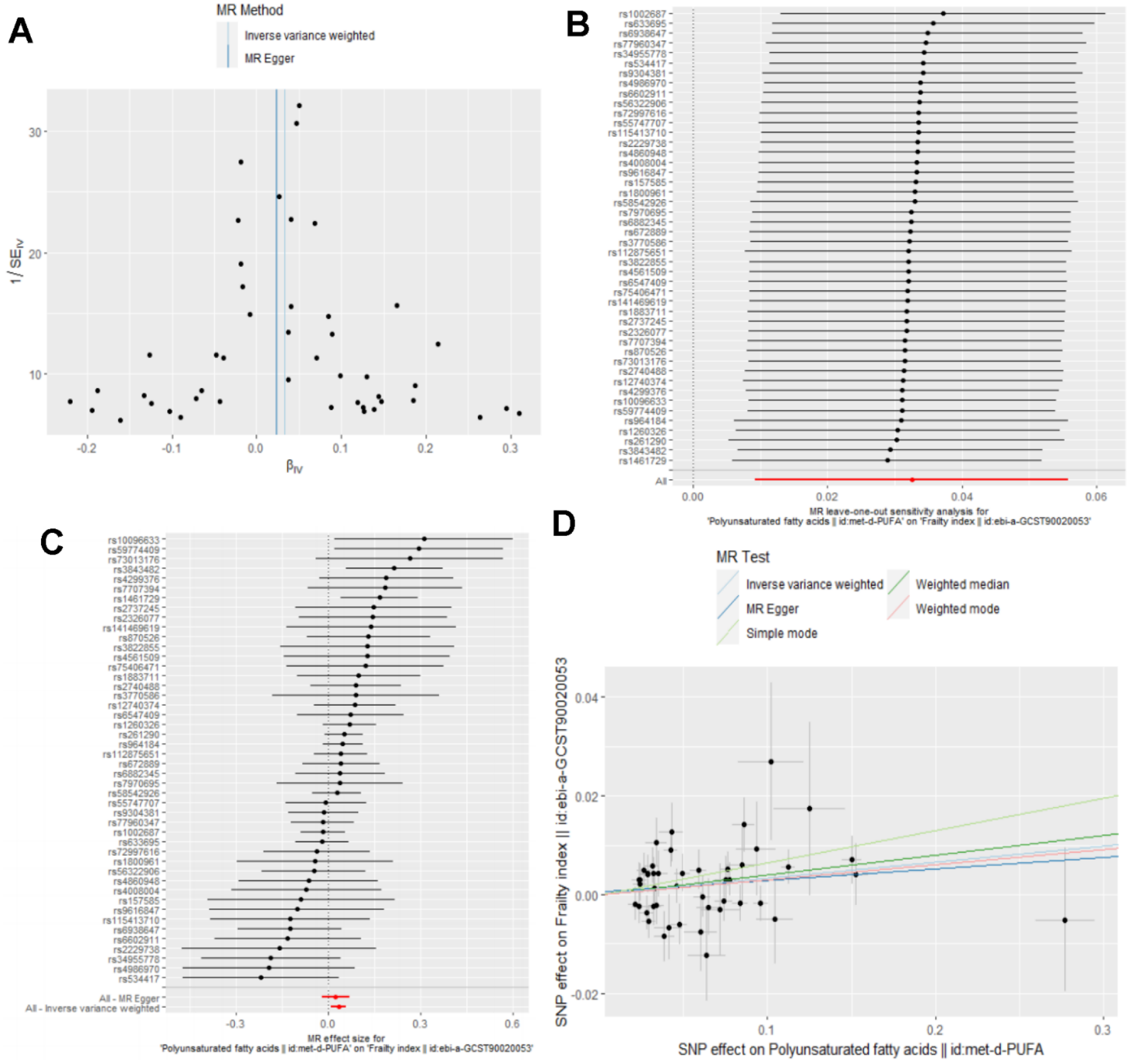
A
MR Method
Inverse variance weighted
MR Egger
1/SE$_{IV}$
$\beta_{IV}$
-0.2 -0.1 0.0 0.1 0.2 0.3
10 20 30
B
All
0.00 0.02 0.04 0.06
MR leave-one-out sensitivity analysis for
'Polyunsaturated fatty acids || id:met-d-PUFA' on 'Frailty index || id:ebi-a-GCST90020053'
C
All - MR Egger
All - Inverse variance weighted
-0.3 0.0 0.3 0.6
MR effect size for
'Polyunsaturated fatty acids || id:met-d-PUFA' on 'Frailty index || id:ebi-a-GCST90020053'
D
MR Test
Inverse variance weighted
MR Egger
Simple mode
Weighted median
Weighted mode
SNP effect on Frailty index || id:ebi-a-GCST90020053
-0.02 0.00 0.02 0.04
0.1 0.2 0.3
SNP effect on Polyunsaturated fatty acids || id:met-d-PUFA

PUFA ON FI

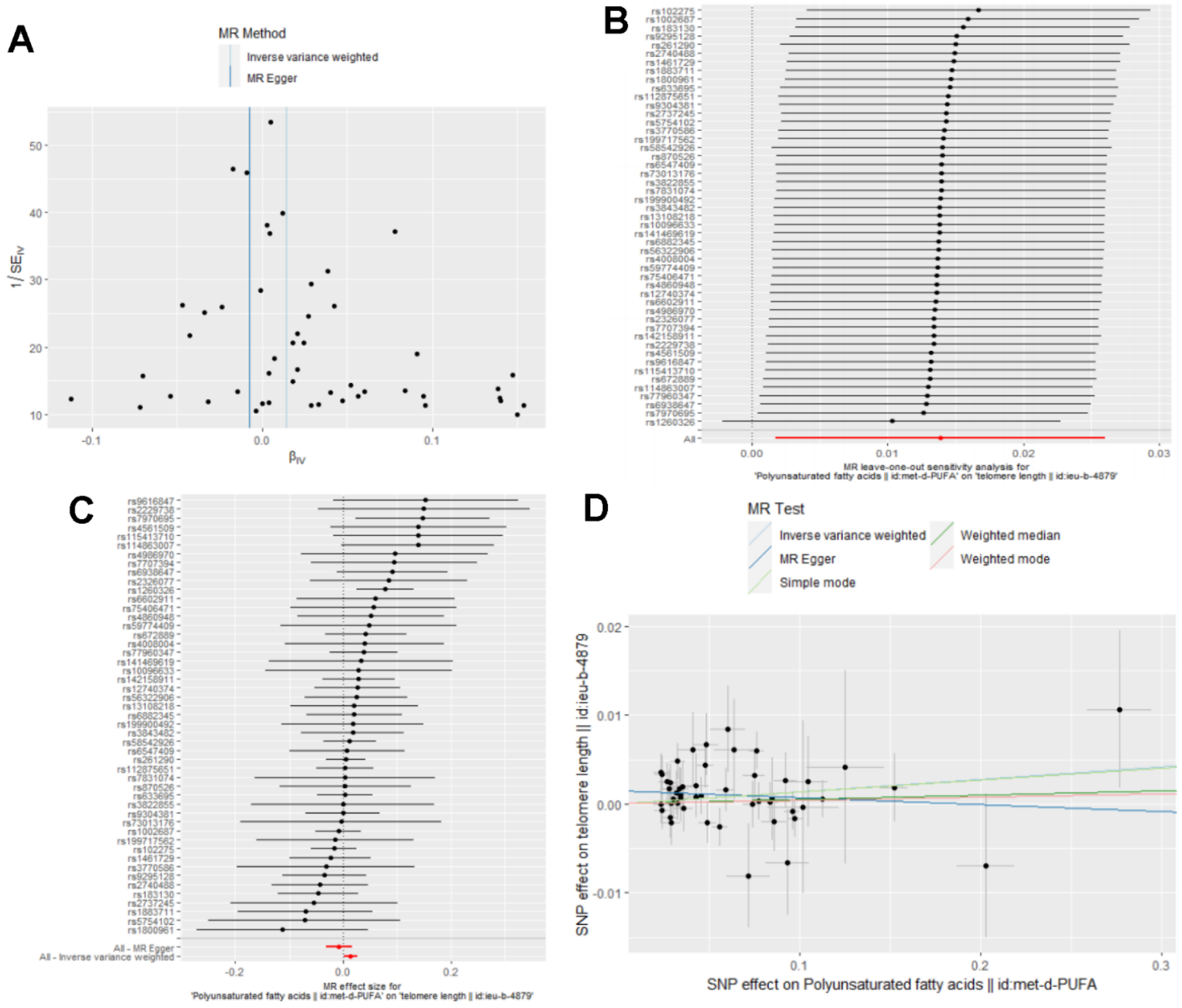

A
MR Method
Inverse variance weighted
MR Egger
$1/SE_{IV}$
$\beta_{IV}$
B
MR leave-one-out sensitivity analysis for
'Polyunsaturated fatty acids || id:met-d-PUFA' on 'telomere length || id:ieu-b-4879'
C
All - MR Egger
All - Inverse variance weighted
MR effect size for
'Polyunsaturated fatty acids || id:met-d-PUFA' on 'telomere length || id:ieu-b-4879'
D
MR Test
Inverse variance weighted
MR Egger
Simple mode
Weighted median
Weighted mode
SNP effect on telomere length || id:ieu-b-4879
SNP effect on Polyunsaturated fatty acids || id:met-d-PUFA


PUFA ON TL

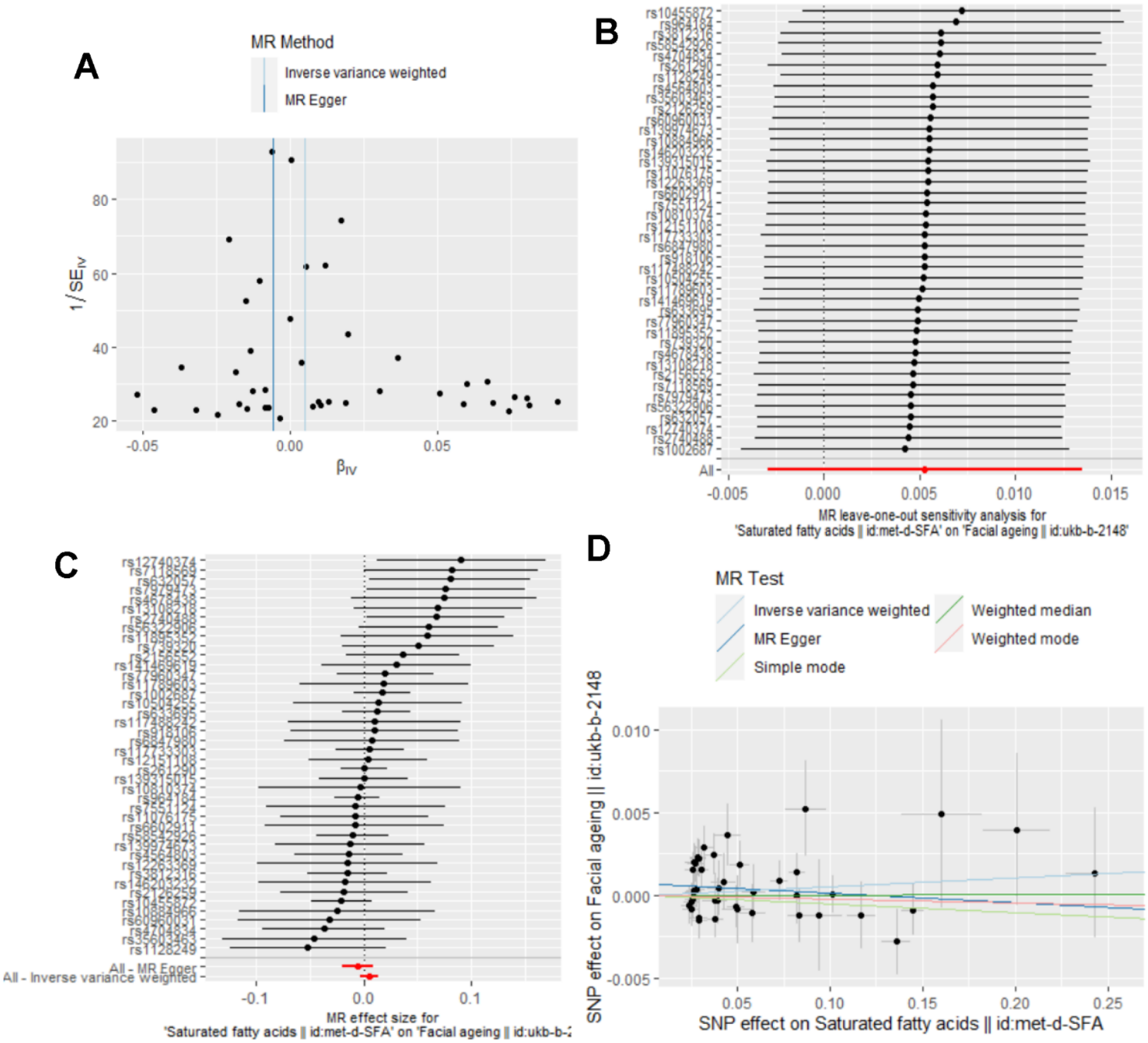
A
MR Method
Inverse variance weighted
MR Egger
1/SE_IV
β_IV
B
MR leave-one-out sensitivity analysis for
'Saturated fatty acids || id:met-d-SFA' on 'Facial ageing || id:ukb-b-2148'
C
All - MR Egger
All - Inverse variance weighted
MR effect size for
D
MR Test
Inverse variance weighted
MR Egger
Simple mode
Weighted median
Weighted mode
SNP effect on Facial ageing || id:ukb-b-2148
SNP effect on Saturated fatty acids || id:met-d-SFA

SFA ON FA

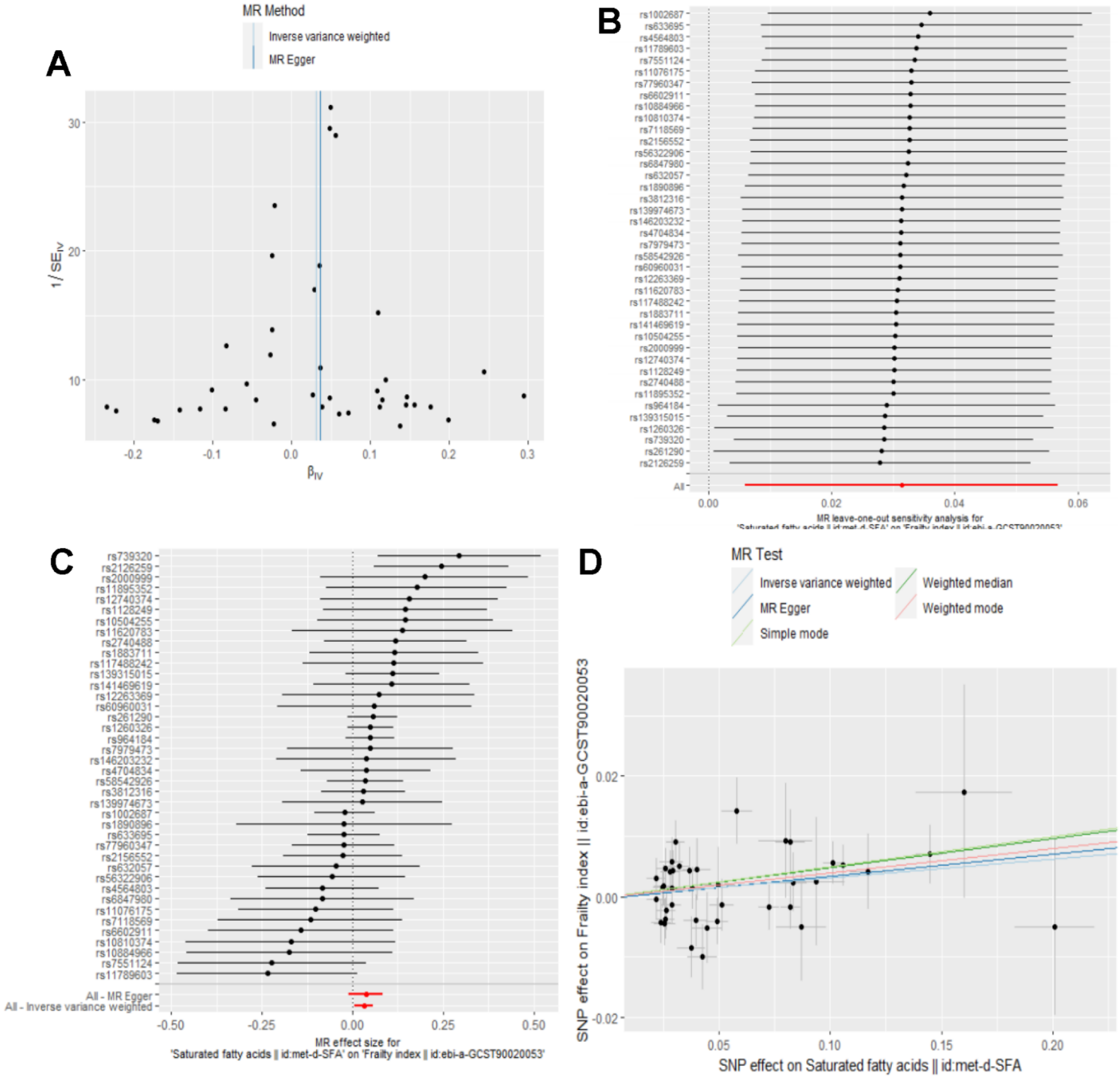
A
MR Method
Inverse variance weighted
MR Egger
1/SE_IV
β_IV
B
MR leave-one-out sensitivity analysis for
All
C
rs739320
rs2126259
rs2000999
rs11895352
rs12740374
rs1128249
rs10504255
rs11620783
rs2740488
rs1883711
rs117488242
rs139315015
rs141469619
rs12263369
rs60960031
rs261290
rs1260326
rs964184
rs7979473
rs146203232
rs4704834
rs58542926
rs3812316
rs139974673
rs1002687
rs1890896
rs633695
rs77960347
rs2156552
rs632057
rs56322906
rs4564803
rs6847980
rs11076175
rs7118569
rs6602911
rs10810374
rs10884966
rs7551124
rs11789603
All - MR Egger
All - Inverse variance weighted
MR effect size for
'Saturated fatty acids || id:met-d-SFA' on 'Frailty index || id:ebi-a-GCST90020053'
D
MR Test
Inverse variance weighted
MR Egger
Simple mode
Weighted median
Weighted mode
SNP effect on Frailty index || id:ebi-a-GCST90020053
SNP effect on Saturated fatty acids || id:met-d-SFA

SFA ON FI

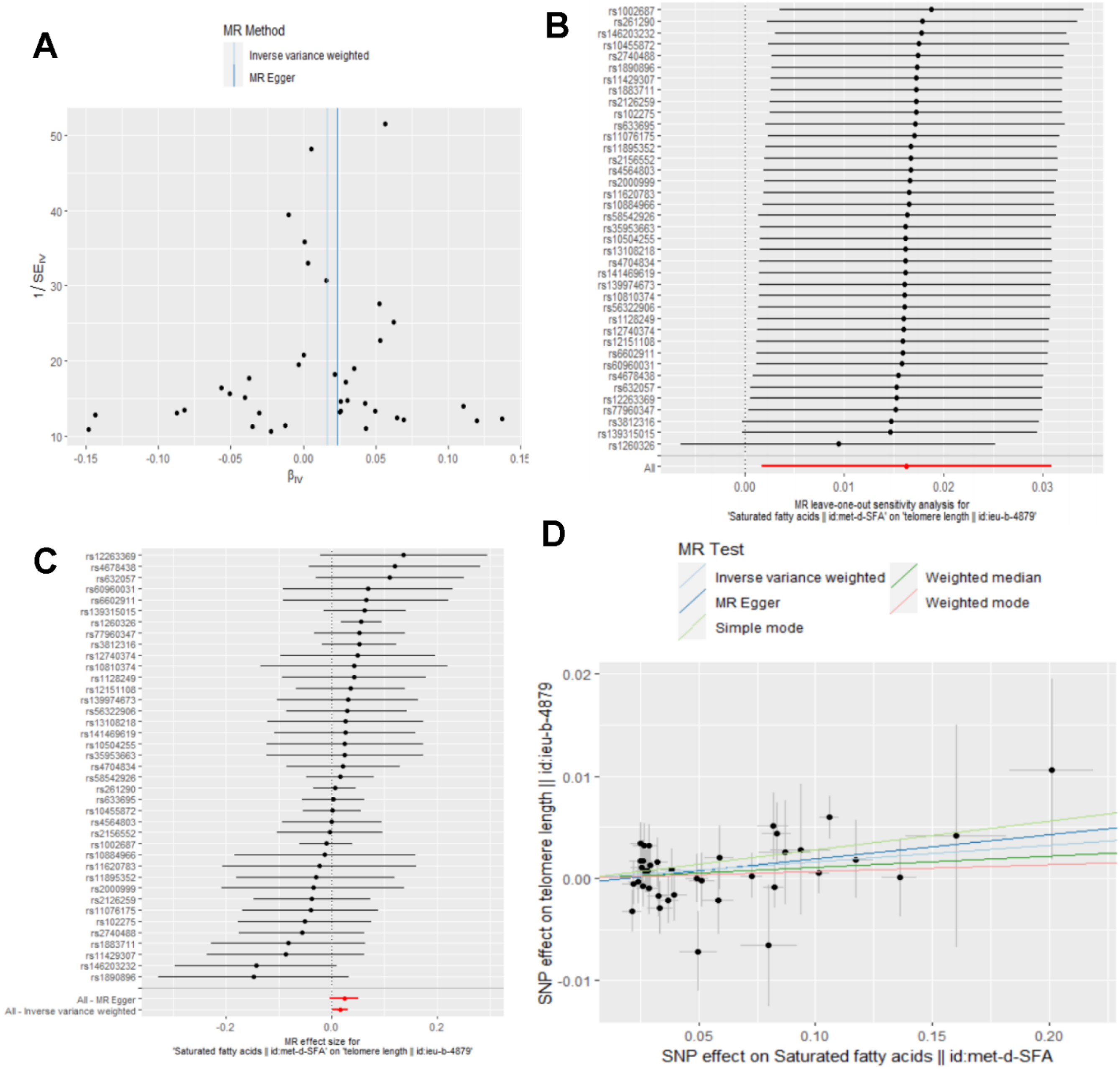
A
MR Method
Inverse variance weighted
MR Egger
1 / SE_IV
β_IV
B
MR leave-one-out sensitivity analysis for
'Saturated fatty acids || id:met-d-SFA' on 'telomere length || id:ieu-b-4879'
C
All - MR Egger
All - Inverse variance weighted
MR effect size for
'Saturated fatty acids || id:met-d-SFA' on 'telomere length || id:ieu-b-4879'
D
MR Test
Inverse variance weighted
MR Egger
Simple mode
Weighted median
Weighted mode
SNP effect on telomere length || id:ieu-b-4879
SNP effect on Saturated fatty acids || id:met-d-SFA

SFA ON TL

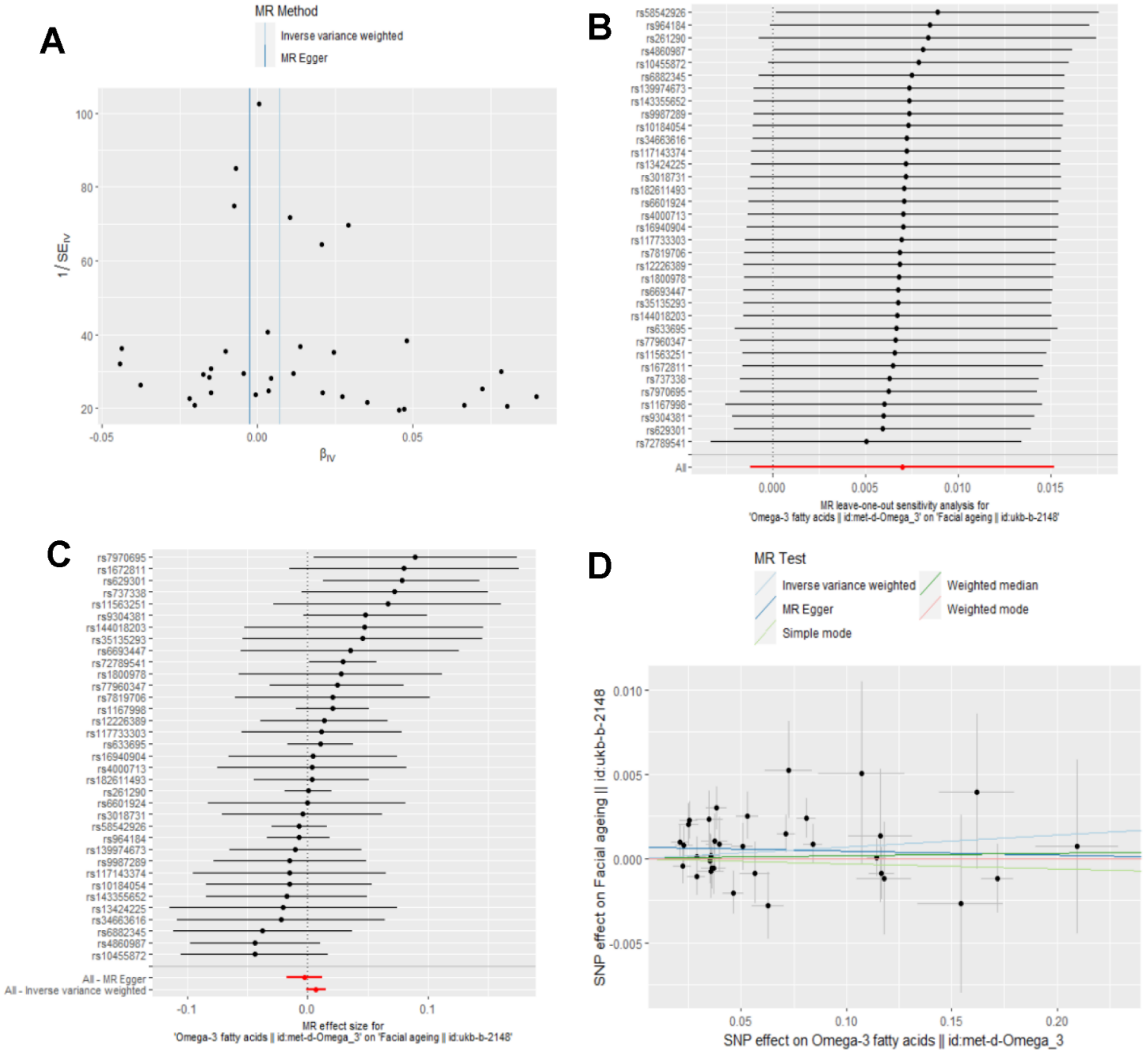
A
MR Method
Inverse variance weighted
MR Egger
1/SE_IV
β_IV
B
MR leave-one-out sensitivity analysis for
'Omega-3 fatty acids || id:met-d-Omega_3' on 'Facial ageing || id:ukb-b-2148'
C
All - MR Egger
All - Inverse variance weighted
MR effect size for
'Omega-3 fatty acids || id:met-d-Omega_3' on 'Facial ageing || id:ukb-b-2148'
D
MR Test
Inverse variance weighted
MR Egger
Simple mode
Weighted median
Weighted mode
SNP effect on Facial ageing || id:ukb-b-2148
SNP effect on Omega-3 fatty acids || id:met-d-Omega_3

Omega-3 ON FA

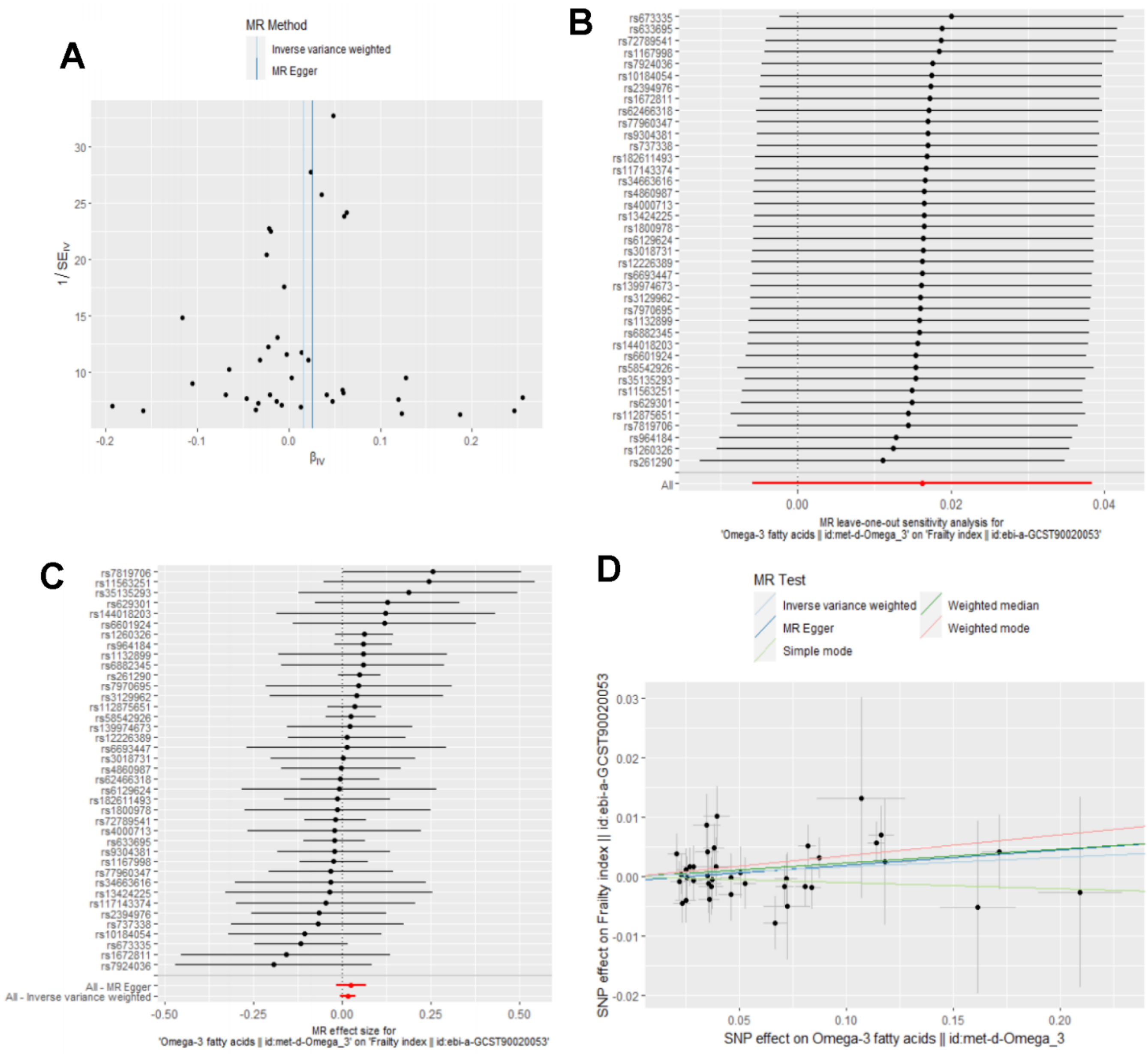
A
MR Method
Inverse variance weighted
MR Egger
1/SE_IV
β_IV
B
MR leave-one-out sensitivity analysis for
'Omega-3 fatty acids || id:met-d-Omega_3' on 'Frailty index || id:ebi-a-GCST90020053'
C
All - MR Egger
All - Inverse variance weighted
MR effect size for
'Omega-3 fatty acids || id:met-d-Omega_3' on 'Frailty index || id:ebi-a-GCST90020053'
D
MR Test
Inverse variance weighted
MR Egger
Simple mode
Weighted median
Weighted mode
SNP effect on Frailty index || id:ebi-a-GCST90020053
SNP effect on Omega-3 fatty acids || id:met-d-Omega_3

Omega-3 ON FI

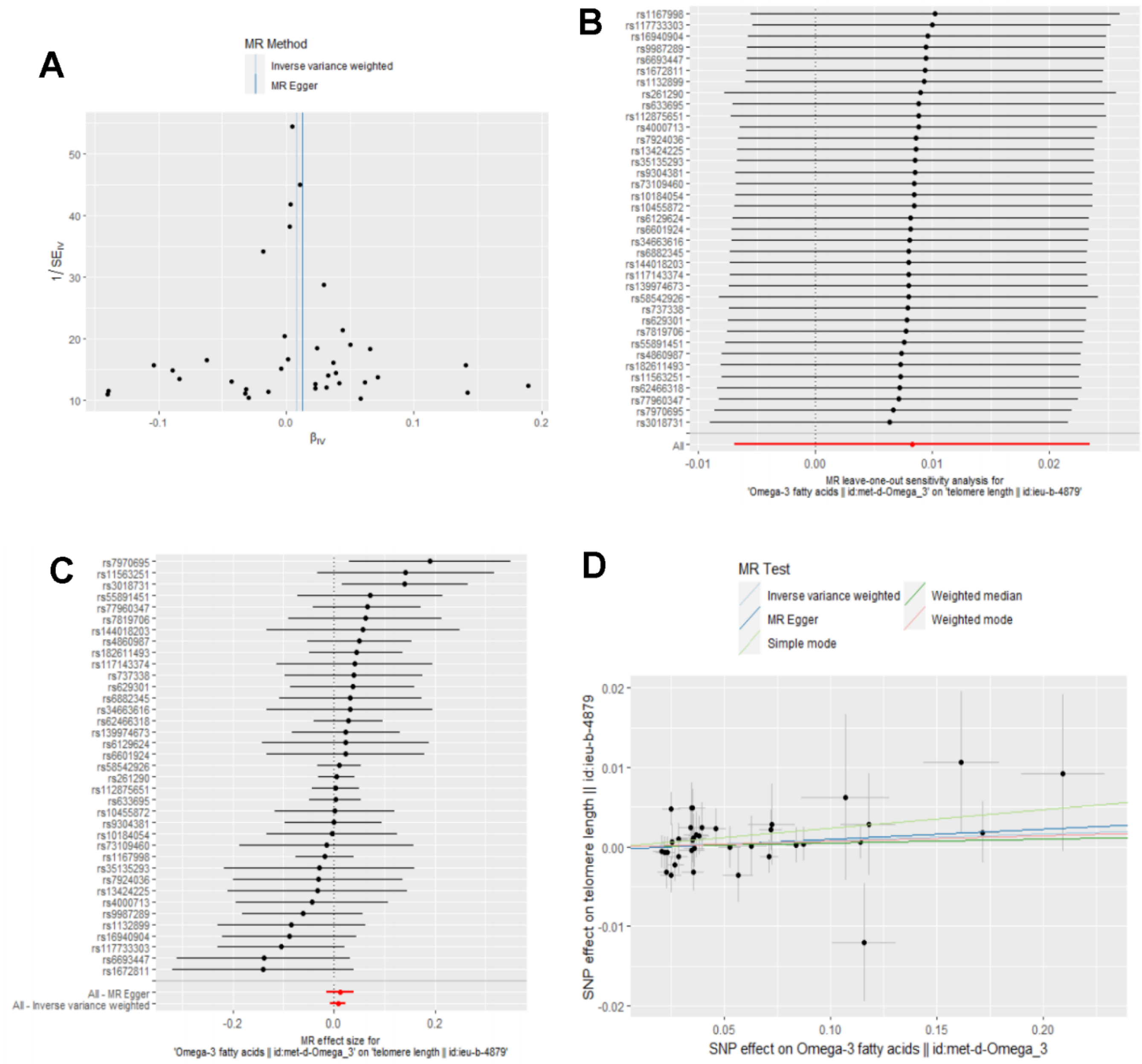
A
MR Method
Inverse variance weighted
MR Egger
1/SE$_{IV}$
$\beta_{IV}$
B
rs1167998
rs117733303
rs16940904
rs9987289
rs6693447
rs1672811
rs1132899
rs261290
rs633695
rs112875651
rs4000713
rs7924036
rs13424225
rs35135293
rs9304381
rs73109460
rs10184054
rs10455872
rs6129624
rs6601924
rs34663616
rs6882345
rs144018203
rs117143374
rs139974673
rs58542926
rs737338
rs629301
rs7819706
rs55891451
rs4860987
rs182611493
rs11563251
rs62466318
rs77960347
rs7970695
rs3018731
All
MR leave-one-out sensitivity analysis for
'Omega-3 fatty acids || id:met-d-Omega_3' on 'telomere length || id:ieu-b-4879'
C
rs7970695
rs11563251
rs3018731
rs55891451
rs77960347
rs7819706
rs144018203
rs4860987
rs182611493
rs117143374
rs737338
rs629301
rs6882345
rs34663616
rs62466318
rs139974673
rs6129624
rs6601924
rs58542926
rs261290
rs112875651
rs633695
rs10455872
rs9304381
rs10184054
rs73109460
rs1167998
rs35135293
rs7924036
rs13424225
rs4000713
rs9987289
rs1132899
rs16940904
rs117733303
rs6693447
rs1672811
All - MR Egger
All - Inverse variance weighted
MR effect size for
'Omega-3 fatty acids || id:met-d-Omega_3' on 'telomere length || id:ieu-b-4879'
D
MR Test
Inverse variance weighted
MR Egger
Simple mode
Weighted median
Weighted mode
SNP effect on telomere length || id:ieu-b-4879
SNP effect on Omega-3 fatty acids || id:met-d-Omega_3

Omega-3 ON TL

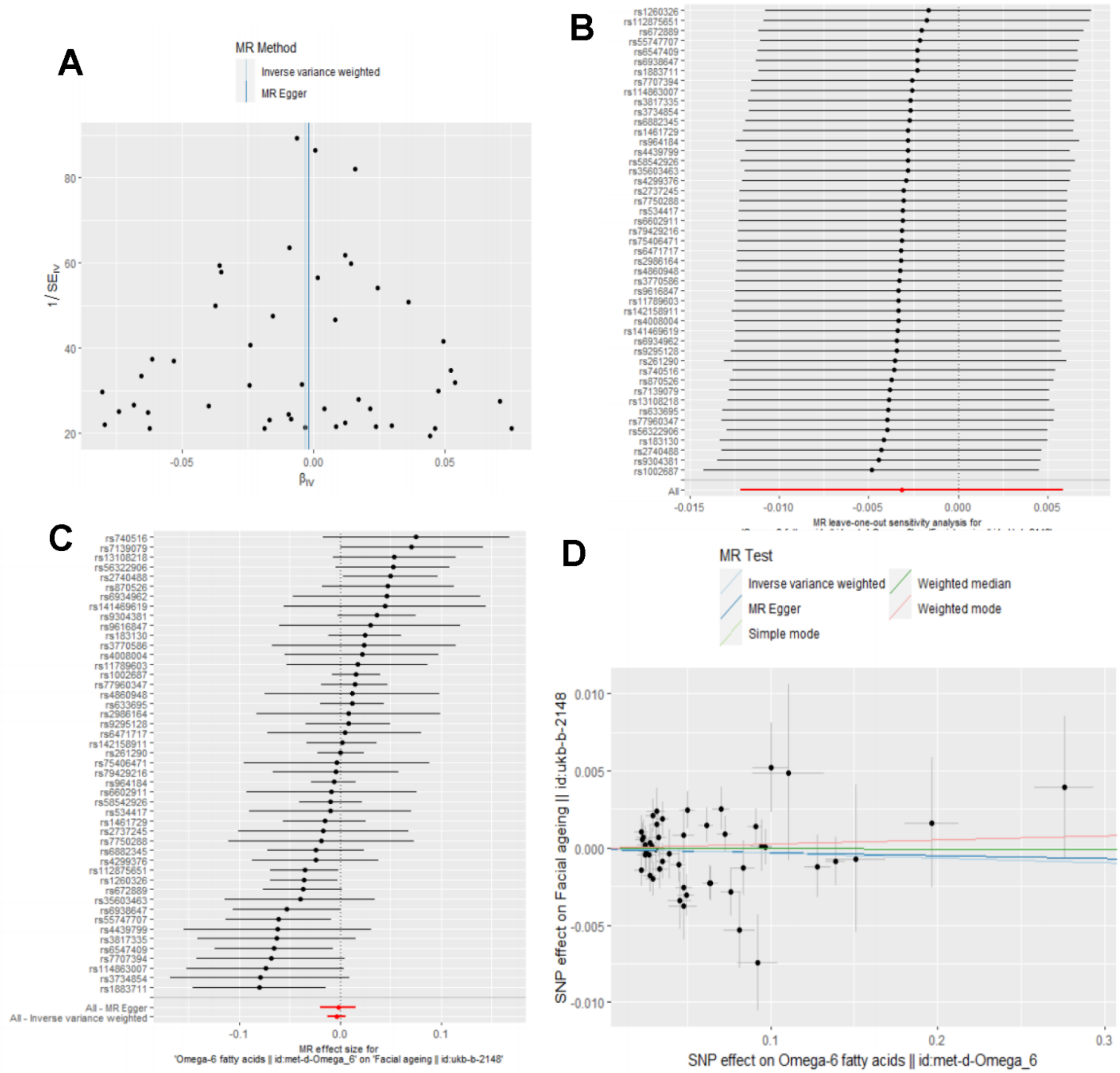
A
MR Method
Inverse variance weighted
MR Egger
1/SE
β
B
MR leave-one-out sensitivity analysis for
C
MR effect size for
'Omega-6 fatty acids || id:met-d-Omega_6' on 'Facial ageing || id:ukb-b-2148'
All - MR Egger
All - Inverse variance weighted
D
MR Test
Inverse variance weighted
MR Egger
Simple mode
Weighted median
Weighted mode
SNP effect on Facial ageing || id:ukb-b-2148
SNP effect on Omega-6 fatty acids || id:met-d-Omega_6

Omega-6 ON FA

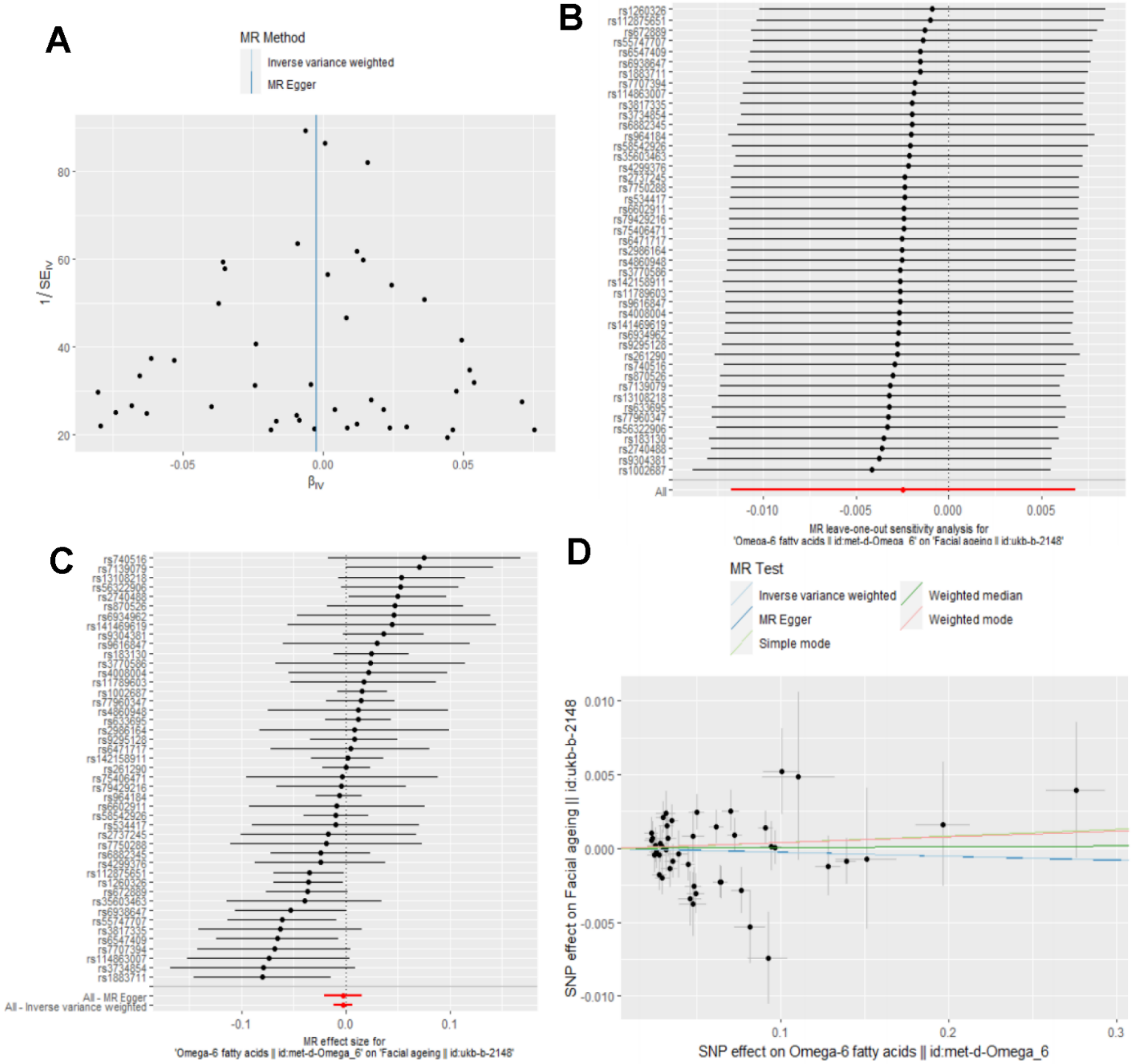
A
MR Method
Inverse variance weighted
MR Egger
1/SE_IV
β_IV
B
MR leave-one-out sensitivity analysis for
'Omega-6 fatty acids || id:met-d-Omega_6' on 'Facial ageing || id:ukb-b-2148'
C
All - MR Egger
All - Inverse variance weighted
MR effect size for
'Omega-6 fatty acids || id:met-d-Omega_6' on 'Facial ageing || id:ukb-b-2148'
D
MR Test
Inverse variance weighted
MR Egger
Simple mode
Weighted median
Weighted mode
SNP effect on Facial ageing || id:ukb-b-2148
SNP effect on Omega-6 fatty acids || id:met-d-Omega_6

Omega-6 ON FI

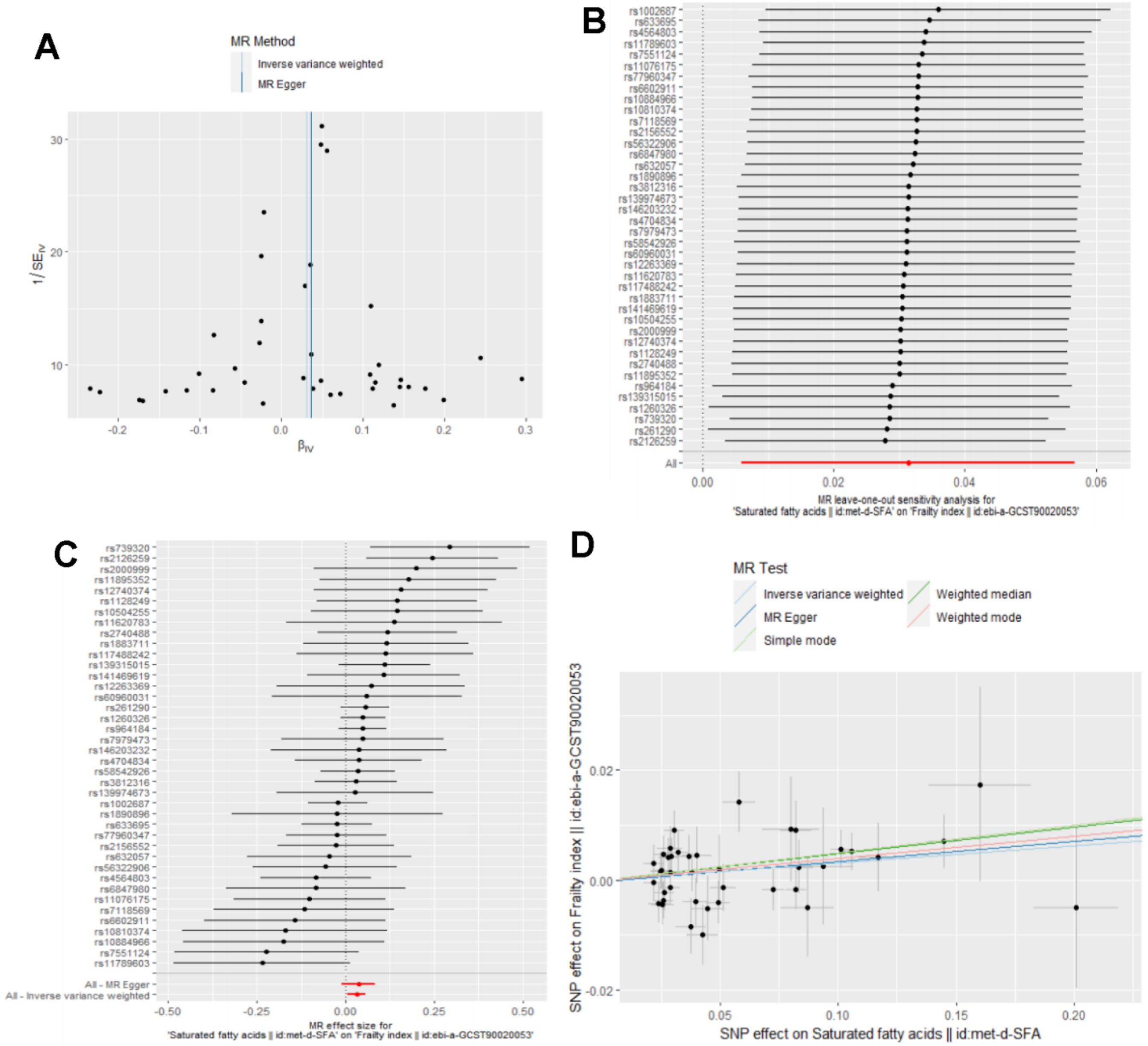


## Omega-6 ON TL

**Supplementary Figure 1.** Main graphical results of MR between five fatty acids (MUFA, PUFA, SFA, Omega-3 fatty acid, and Omega-6 fatty acid) and aging proxies (TL, FI, and FA), (**A**) funnel plots; (**B**) leave-one-out plots; (**C**) IVW graphical results; (**D**) scatter plots of the five MR analyses.
