# Supplementary material for: Relationship between fatty acid intake and aging: a Mendelian randomization study
Source: Aging (Albany NY). 2024 Mar 26;16(6):5711–39. doi: 10.18632/aging.205674 (PMC11006485; doi:10.18632/aging.205674)
Supplement: Supplementary Table 1 [file aging-16-205674-s002.pdf]

SUPPLEMENTARY TABLE

Supplementary Table 1. Summary-level genome-wide association studies (GWAS) used in this article.

Exposure	GWAS ID	Consortium	Sample size
Saturated FA	met-d-SFA	UK biobank	114,999
Monounsaturated FA	met-d-MUFA	UK biobank	114,999
Polyunsaturated FA	met-d-PUFA	UK biobank	114,999
Omega-3 FA	met-d-Omega_3	UK biobank	114,999
Omega-6 FA	met-d-Omega_6	UK biobank	114,999
outcome			
telomere length	ieu-b-4879	UK biobank	472,174
Facial ageing	ukb-b-2148	MRC-IEU	423,999
Frailty index	ebi-a-GCST90020053	UK biobank	175,226
