# Supplementary material for: Relationship between fatty acid intake and aging: a Mendelian randomization study
Source: Aging (Albany NY). 2024 Mar 26;16(6):5711–39. doi: 10.18632/aging.205674 (PMC11006485; doi:10.18632/aging.205674)
Supplement: Supplementary Table 2 [file aging-16-205674-s003.docx]

| Exposure | Outcome | No.SNP | Methods | OR | (95% CI) | P-value | Heterogeneity | | | | Pleiotropy | |
| --- | --- | --- | --- | --- | --- | --- | --- | --- | --- | --- | --- | --- |
|  |  |  |  |  |  |  | MR-Egger | | IVW | | MR-Egger | |
|  |  |  |  |  |  |  | Q | P | Q | P | Intercept | P |
| TL | MUFA | 147 | IVW | 1.037006 | 0.9909609-1.085191 | 0.1168468 | 325.4598 | p＜0.001 | 326.4311 | p＜0.001 | 0.0007552 | 0.511692 |
| TL | PUFA | 147 | IVW | 1.0201838 | 0.9628635-1.080917 | 0.4982104 | 542.5295 | p＜0.001 | 545.1247 | p＜0.001 | 0.00121638 | 0.406307 |
| TL | SFA | 147 | IVW | 1.0190220 | 0.9756295-1.064344 | 0.3960343 | 298.2629 | p＜0.001 | 299.1406 | p＜0.001 | 0.00071863 | 0.514642 |
| TL | Omega-6 FA | 147 | IVW | 1.0185058 | 0.9606460-1.079851 | 0.5388827 | 553.8375 | p＜0.001 | 556.2734 | p＜0.001 | 0.0011798 | 0.425834 |
| FI | MUFA | 15 | IVW | 1.340525 | 1.1778700-1.525642 | p＜0.001 | 22.32916 | 0.050466 | 24.13049 | 0.0441866 | 0.0067473 | 0.324478 |
| FI | PUFA | 15 | IVW | 1.0465042 | 0.9183988-1.192479 | 0.4950571 | 24.48707 | 0.026934 | 25.32197 | 0.0315186 | -0.0045272 | 0.517199 |
| FI | SFA | 15 | IVW | 1.304578 | 1.1566157-1.471469 | p＜0.001 | 20.85403 | 0.075866 | 20.85645 | 0.1053680 | 0.00024783 | 0.969577 |

Supplementary Table 2. Inverse MR results for the TL and FI.
