# Supplementary material for: Relationship between fatty acid intake and aging: a Mendelian randomization study
Source: Aging (Albany NY). 2024 Mar 26;16(6):5711–39. doi: 10.18632/aging.205674 (PMC11006485; doi:10.18632/aging.205674)
Supplement: Supplementary Table 3 [file aging-16-205674-s004.docx]

| Exposure | Outcome | No.SNP | F | R^2^ | Methods | OR | 95%CI | Pvalue | Heterogeneity | | | | Pleiotropy | | | |
| --- | --- | --- | --- | --- | --- | --- | --- | --- | --- | --- | --- | --- | --- | --- | --- | --- |
|  |  |  |  |  |  |  |  |  | MR-Egger | | IVW | | MR-Egger | | MR-PRESSO | |
|  |  |  |  |  |  |  |  |  | Q | P | Q | P | Intercept | P | No. of outlier | Global-P |
| MUFA | TL | 49 | 406.85685 | 0.0185 | MR Egger | 1.026977 | 1.0033318-1.051179 | 0.029866612 | 36.74529 | 0.8590417 | 37.29683 | 0.8679291 | -0.0004732078 | 0.4613834 | NA | 0.862 |
|  |  |  |  |  | Weighted median | 1.019029 | 0.9995508-1.038886 | 0.055572981 |  |  |  |  |  |  |  |  |
|  |  |  |  |  | Inverse variance weighted | 1.019485 | 1.0063093-1.032834 | 0.003641659 |  |  |  |  |  |  |  |  |
|  |  |  |  |  | Simple mode | 1.021335 | 0.9877392-1.056073 | 0.222077282 |  |  |  |  |  |  |  |  |
|  |  |  |  |  | Weighted mode | 1.022195 | 0.9965934-1.048454 | 0.096303508 |  |  |  |  |  |  |  |  |
| MUFA | FA | 39 | 100.30534 | 0.0000684 | MR Egger | 0.9892913 | 0.9770351-1.001701 | 0.09890824 | 51.57018 | 0.05625315 | 52.57376 | 0.05817208 | 0.0003298611 | 0.4015869 | NA | 0.082 |
|  |  |  |  |  | Weighted median | 0.9955387 | 0.9853849-1.005797 | 0.39262697 |  |  |  |  |  |  |  |  |
|  |  |  |  |  | Inverse variance weighted | 0.9933550 | 0.9853622-1.001413 | 0.10576794 |  |  |  |  |  |  |  |  |
|  |  |  |  |  | Simple mode | 0.9885094 | 0.9719949-1.005304 | 0.18674830 |  |  |  |  |  |  |  |  |
|  |  |  |  |  | Weighted mode | 0.9933527 | 0.9838587-1.002938 | 0.18146817 |  |  |  |  |  |  |  |  |
| MUFA | FI | 48 | 620.78481 | 0.00153 | MR Egger | 1.020161 | 0.9863254-1.055157 | 0.2520790386 | 37.57710 | 0.8071996 | 39.39767 | 0.7767001 | 0.001313729 | 0.1838486 | NA | 0.788 |
|  |  |  |  |  | Weighted median | 1.035090 | 1.0037858-1.067371 | 0.0277244027 |  |  |  |  |  |  |  |  |
|  |  |  |  |  | Inverse variance weighted | 1.039051 | 1.0177932-1.060754 | 0.0002809507 |  |  |  |  |  |  |  |  |
|  |  |  |  |  | Simple mode | 1.026276 | 0.9717025-1.083915 | 0.3569444176 |  |  |  |  |  |  |  |  |
|  |  |  |  |  | Weighted mode | 1.037777 | 1.0059286-1.070633 | 0.0240538148 |  |  |  |  |  |  |  |  |

Supplementary Table 3. Main result of Mendelian randomization study.

| Exposure | Outcome | No.SNP | F | R^2^ | Methods | OR | 95%CI | Pvalue | Heterogeneity | | | | Pleiotropy | | | | | |
| --- | --- | --- | --- | --- | --- | --- | --- | --- | --- | --- | --- | --- | --- | --- | --- | --- | --- | --- |
|  |  |  |  |  |  |  |  |  | MR-Egger | | IVW | | MR-Egger | | | MR-PRESSO | | |
|  |  |  |  |  |  |  |  |  | Q | P | Q | P | Intercept | P | | No. of outlier | | Global-P |
| PUFA | TL | 49 | 242.13894 | 0.0193 | MR Egger | 0.9923318 | 0.9689307-1.016298 | 0.53030392 | 42.38562 | 0.6638750 | 46.62696 | 0.5291859 | 0.001483442 | | 0.04501461 | | NA | 0.527 |
|  |  |  |  |  | Weighted median | 1.0049015 | 0.9873056-1.022811 | 0.58747157 |  |  |  |  |  | |  | |  |  |
|  |  |  |  |  | Inverse variance weighted | 1.0139840 | 1.0017431-1.026374 | 0.02502313 |  |  |  |  |  | |  | |  |  |
|  |  |  |  |  | Simple mode | 1.0135098 | 0.9830349-1.044929 | 0.39324071 |  |  |  |  |  | |  | |  |  |
|  |  |  |  |  | Weighted mode | 1.0037445 | 0.9827683-1.025168 | 0.73021161 |  |  |  |  |  | |  | |  |  |
| PUFA | FA | 43 | 45.715546 | 0.00976 | MR Egger | 1.002558 | 0.9871561-1.018201 | 0.7479828 | 64.21199 | 0.01172937 | 64.34409 | 0.01484118 | 0.0001325438 | | 0.7729572 | | NA | 0.018 |
|  |  |  |  |  | Weighted median | 1.001346 | 0.9906356-1.012172 | 0.8063273 |  |  |  |  |  | |  | |  |  |
|  |  |  |  |  | Inverse variance weighted | 1.004476 | 0.9960011-1.013023 | 0.3015577 |  |  |  |  |  | |  | |  |  |
|  |  |  |  |  | Simple mode | 1.007377 | 0.9873610-1.027799 | 0.4768582 |  |  |  |  |  | |  | |  |  |
|  |  |  |  |  | Weighted mode | 1.002660 | 0.9913751-1.014074 | 0.6478581 |  |  |  |  |  | |  | |  |  |
| PUFA | FI | 46 | 336.6762 | 0.00133 | MR Egger | 1.023586 | 0.9791149-1.070078 | 0.309253690 | 57.54495 | 0.08269093 | 57.84532 | 0.09477001 | 0.0006429804 | | 0.6341472 | | NA | 0.105 |
|  |  |  |  |  | Weighted median | 1.040929 | 1.0092906-1.073558 | 0.010857859 |  |  |  |  |  | |  | |  |  |
|  |  |  |  |  | Inverse variance weighted | 1.033057 | 1.0092288-1.057449 | 0.006303351 |  |  |  |  |  | |  | |  |  |
|  |  |  |  |  | Simple mode | 1.067679 | 1.0153145-1.122744 | 0.014163816 |  |  |  |  |  | |  | |  |  |
|  |  |  |  |  | Weighted mode | 1.030906 | 0.9987852-1.064060 | 0.065933578 |  |  |  |  |  | |  | |  |  |

| Exposure | Outcome | No.SNP | F | R^2^ | Methods | OR | 95%CI | Pvalue | Heterogeneity | | | | Pleiotropy | | | |
| --- | --- | --- | --- | --- | --- | --- | --- | --- | --- | --- | --- | --- | --- | --- | --- | --- |
|  |  |  |  |  |  |  |  |  | MR-Egger | | IVW | | MR-Egger | | MR-PRESSO | |
|  |  |  |  |  |  |  |  |  | Q | P | Q | P | Intercept | P | No. of outlier | Global-P |
| SFA | TL | 39 | 183.052 | 0.0159 | MR Egger | 1.023923 | 0.9966152-1.051980 | 0.09486481 | 32.10078 | 0.6978816 | 32.50260 | 0.7211745 | -0.0004631121 | 0.5300487 | NA | 0.7 |
|  |  |  |  |  | Weighted median | 1.010985 | 0.9890201-1.033438 | 0.32963165 |  |  |  |  |  |  |  |  |
|  |  |  |  |  | Inverse variance weighted | 1.016415 | 1.0016930-1.031354 | 0.02872505 |  |  |  |  |  |  |  |  |
|  |  |  |  |  | Simple mode | 1.028553 | 0.9883715-1.070369 | 0.17422001 |  |  |  |  |  |  |  |  |
|  |  |  |  |  | Weighted mode | 1.006635 | 0.9761736-1.038046 | 0.67554706 |  |  |  |  |  |  |  |  |
| SFA | FA | 42 | 66.333979 | 0.00116 | MR Egger | 0.9943234 | 0.9803658-1.008480 | 0.4346025 | 45.48422 | 0.2543095 | 49.33511 | 0.1743508 | 0.0007210889 | 0.07315723 | NA | 0.188 |
|  |  |  |  |  | Weighted median | 1.0002928 | 0.9889785-1.011737 | 0.9597723 |  |  |  |  |  |  |  |  |
|  |  |  |  |  | Inverse variance weighted | 1.0052922 | 0.9970974-1.013554 | 0.2062564 |  |  |  |  |  |  |  |  |
|  |  |  |  |  | Simple mode | 0.9947685 | 0.9752198-1.014709 | 0.6072477 |  |  |  |  |  |  |  |  |
|  |  |  |  |  | Weighted mode | 0.9977194 | 0.9851504-1.010449 | 0.7259119 |  |  |  |  |  |  |  |  |
| SFA | FI | 40 | 229.3927 | 0.00417 | MR Egger | 1.037315 | 0.9897896-1.087122 | 0.13403104 | 45.51631 | 0.1876764 | 45.60006 | 0.2166816 | -0.0003449662 | 0.792881 | NA | 0.25 |
|  |  |  |  |  | Weighted median | 1.049554 | 1.0139070-1.086453 | 0.006080319 |  |  |  |  |  |  |  |  |
|  |  |  |  |  | Inverse variance weighted | 1.031839 | 1.0059763-1.058366 | 0.015516952 |  |  |  |  |  |  |  |  |
|  |  |  |  |  | Simple mode | 1.051257 | 0.9879333-1.118640 | 0.122873622 |  |  |  |  |  |  |  |  |
|  |  |  |  |  | Weighted mode | 1.041019 | 1.0016693-1.081915 | 0.047661249 |  |  |  |  |  |  |  |  |

| Exposure | Outcome | No.SNP | F | R^2^ | Methods | OR | 95%CI | Pvalue | Heterogeneity | | | | Pleiotropy | | | |
| --- | --- | --- | --- | --- | --- | --- | --- | --- | --- | --- | --- | --- | --- | --- | --- | --- |
|  |  |  |  |  |  |  |  |  | MR-Egger | | IVW | | MR-Egger | | MR-PRESSO | |
|  |  |  |  |  |  |  |  |  | Q | P | Q | P | Intercept | P | No. of outlier | Global-P |
| Omega_6 | TL | 40 | 229.39273 | 0.000638 | MR Egger | 1.037315 | 0.9897896-1.087122 | 0.134031041 | 45.51631 | 0.1876764 | 45.60006 | 0.2166816 | -0.0003449662 | 0.792881 | NA | 0.265 |
|  |  |  |  |  | Weighted median | 1.049554 | 1.0147281-1.085574 | 0.004965994 |  |  |  |  |  |  |  |  |
|  |  |  |  |  | Inverse variance weighted | 1.031839 | 1.0059763-1.058366 | 0.015516952 |  |  |  |  |  |  |  |  |
|  |  |  |  |  | Simple mode | 1.051257 | 0.9859953-1.120839 | 0.134413845 |  |  |  |  |  |  |  |  |
|  |  |  |  |  | Weighted mode | 1.041019 | 1.0064411-1.076785 | 0.024926071 |  |  |  |  |  |  |  |  |
| Omega_6 | FA | 47 | 22.508787 | 0.0000614 | MR Egger | 0.9980587 | 0.9807975-1.015624 | 0.8281713 | 79.58167 | 0.001127763 | 79.62627 | 0.001528551 | -7.830386e-05 | 0.8745267 | NA | 0.002 |
|  |  |  |  |  | Weighted median | 0.9995965 | 0.9886327-1.010682 | 0.9428248 |  |  |  |  |  |  |  |  |
|  |  |  |  |  | Inverse variance weighted | 0.9968564 | 0.9879069-1.005887 | 0.4937943 |  |  |  |  |  |  |  |  |
|  |  |  |  |  | Simple mode | 1.0026833 | 0.9787529-1.027199 | 0.8288357 |  |  |  |  |  |  |  |  |
|  |  |  |  |  | Weighted mode | 1.0026833 | 0.9890836-1.016470 | 0.7023036 |  |  |  |  |  |  |  |  |
| Omega_6 | FI | 45 | 12.88056 | 0.0000354 | MR Egger | 0.9973018 | 0.9794794-1.015448 | 0.7704168 | 77.67983 | 0.0009373286 | 77.68154 | 0.0012974817 | 1.579555e-05 | 0.9755746 | NA | 0.004 |
|  |  |  |  |  | Weighted median | 1.0006135 | 0.9890173-1.012346 | 0.9178702 |  |  |  |  |  |  |  |  |
|  |  |  |  |  | Inverse variance weighted | 0.9975432 | 0.9883461-1.006826 | 0.6027119 |  |  |  |  |  |  |  |  |
|  |  |  |  |  | Simple mode | 1.0044525 | 0.9793884-1.030158 | 0.7320482 |  |  |  |  |  |  |  |  |
|  |  |  |  |  | Weighted mode | 1.0039630 | 0.9910223-1.017073 | 0.5532080 |  |  |  |  |  |  |  |  |

| Exposure | Outcome | No.SNP | F | R^2^ | Methods | OR | 95%CI | Pvalue | Heterogeneity | | | | Pleiotropy | | | |
| --- | --- | --- | --- | --- | --- | --- | --- | --- | --- | --- | --- | --- | --- | --- | --- | --- |
|  |  |  |  |  |  |  |  |  | MR-Egger | | IVW | | MR-Egger | | MR-PRESSO | |
|  |  |  |  |  |  |  |  |  | Q | P | Q | P | Intercept | P | No. of outlier | Global-P |
| Omega_3 | TL | 37 | 41.576721 | 0.000107 | MR Egger | 1.012632 | 0.9850912-1.040943 | 0.3783336 | 31.94904 | 0.6161748 | 32.08154 | 0.6555318 | -0.0002801547 | 0.7180451 | NA | 0.69 |
|  |  |  |  |  | Weighted median | 1.004861 | 0.9830311-1.027175 | 0.6652257 |  |  |  |  |  |  |  |  |
|  |  |  |  |  | Inverse variance weighted | 1.008313 | 0.9931186-1.023739 | 0.2852372 |  |  |  |  |  |  |  |  |
|  |  |  |  |  | Simple mode | 1.023576 | 0.9887202-1.059661 | 0.1957445 |  |  |  |  |  |  |  |  |
|  |  |  |  |  | Weighted mode | 1.007172 | 0.9854654-1.029357 | 0.5243780 |  |  |  |  |  |  |  |  |
| Omega_3 | FA | 35 | 95.325001 | 0.000219 | MR Egger | 0.9976048 | 0.9830367-1.012389 | 0.75135897 | 33.91357 | 0.4233140 | 36.20953 | 0.3658437 | 0.0006687839 | 0.1444962 | NA | 0.372 |
|  |  |  |  |  | Weighted median | 1.0014910 | 0.9888933-1.014249 | 0.81755552 |  |  |  |  |  |  |  |  |
|  |  |  |  |  | Inverse variance weighted | 1.0070080 | 0.9987730-1.015311 | 0.09552477 |  |  |  |  |  |  |  |  |
|  |  |  |  |  | Simple mode | 0.9970394 | 0.9755544-1.018998 | 0.79125874 |  |  |  |  |  |  |  |  |
|  |  |  |  |  | Weighted mode | 0.9999496 | 0.9875616-1.012493 | 0.99372972 |  |  |  |  |  |  |  |  |
| Omega_3 | FI | 39 | 79.40497 | 0.000212 | MR Egger | 1.0261288 | 0.9842025-1.069841 | 0.23324954 | 26.38451 | 0.9027234 | 26.66579 | 0.9160950 | -0.0006661901 | 0.5990269 | NA | 0.907 |
|  |  |  |  |  | Weighted median | 1.0236357 | 0.9911537-1.057182 | 0.15563295 |  |  |  |  |  |  |  |  |
|  |  |  |  |  | Inverse variance weighted | 1.0163515 | 0.9941359-1.039063 | 0.15031821 |  |  |  |  |  |  |  |  |
|  |  |  |  |  | Simple mode | 0.9897128 | 0.9360513-1.046451 | 0.71819014 |  |  |  |  |  |  |  |  |
|  |  |  |  |  | Weighted mode | 1.0358581 | 0.9980018-1.075150 | 0.07141137 |  |  |  |  |  |  |  |  |
